# Supplementary material for: FABIO: TWAS fine-mapping to prioritize causal genes for binary traits
Source: PLoS Genet. 2024 Dec 2;20(12):e1011503. doi: 10.1371/journal.pgen.1011503 (PMC11649093; doi:10.1371/journal.pgen.1011503)
Supplement: S1 Table — The table summarizes the number of discoveries for each of the six disease traits (rows) in the TWAS fine-mapping analysis of UK Biobank. A risk region with GWAS or TWAS signals (1st column) is defined as an LD block that harbors at least one genome-wide significant SNP or significant TWAS gene same as the definition we applied in Table 1 of the main text. The following two columns list the number of genes discovered by applying FABIO with default prior and beta prior on π, respectively. The number in the bracket is the number of identified genes that are located in a risk region with GWAS or TWAS signals. We used an estimated FDR threshold of 0.05 to declare significance in the fine-mapping analysis. (DOCX) [file pgen.1011503.s001.docx]

S1 Table. Summary results of TWAS fine-mapping in UK Biobank (FABIO input options for $\pi$)

| Trait | Risk regions with GWAS or TWAS signals | default prior on $\pi$ | beta prior on $\pi$ |
| --- | --- | --- | --- |
| AS | 24 | 23 (15) | 8 (3) |
| BRCA | 18 | 12 (2) | 5 (1) |
| GO | 33 | 34 (16) | 14 (6) |
| HT | 228 | 254 (159) | 77 (38) |
| PRCA | 19 | 21(7) | 8 (1) |
| RA | 11 | 20 (14) | 6 (3) |
