# Supplementary material for: FABIO: TWAS fine-mapping to prioritize causal genes for binary traits
Source: PLoS Genet. 2024 Dec 2;20(12):e1011503. doi: 10.1371/journal.pgen.1011503 (PMC11649093; doi:10.1371/journal.pgen.1011503)
Supplement: S2 Table — The table summarizes the number of discoveries for each of the six disease traits (rows) in the subset TWAS fine-mapping analysis of UK Biobank. We covered 8,411 genes from 1,259 unique LD blocks that contain no more than 20 genes in each LD block. A risk region with GWAS or TWAS signals (1st column) is defined as an LD block that harbors at least one genome-wide significant SNP or significant TWAS gene same as the definition we applied in Table 1 of the main text. The following five columns list the number of genes discovered by each of the five methods. The number in the bracket is the number of identified genes that are located in a risk region with GWAS or TWAS signals. We used an estimated FDR threshold of 0.05 to declare significance for all methods in the fine-mapping analysis. (DOCX) [file pgen.1011503.s002.docx]

S2 Table. Summary results of TWAS fine-mapping in UK Biobank (five methods)

| Trait | Risk regions with GWAS or TWAS signals | FABIO | FOCUS | FOGS | GIFT | cTWAS |
| --- | --- | --- | --- | --- | --- | --- |
| AS | 18 | 20 (13) | 48 (9) | 65 (8) | 15 (10) | 16 (9) |
| BRCA | 14 | 8 (1) | 10 (1) | 52 (3) | 7 (1) | 10 (1) |
| GO | 30 | 28 (14) | 36 (12) | 69 (8) | 24 (10) | 22 (10) |
| HT | 205 | 210 (141) | 202 (118) | 192 (95) | 195 (125) | 185 (122) |
| PRCA | 16 | 18(5) | 15 (1) | 57 (0) | 12 (3) | 10 (2) |
| RA | 10 | 18 (12) | 20 (10) | 75 (13) | 16 (10) | 18 (10) |
