# Supplementary material for: FABIO: TWAS fine-mapping to prioritize causal genes for binary traits
Source: PLoS Genet. 2024 Dec 2;20(12):e1011503. doi: 10.1371/journal.pgen.1011503 (PMC11649093; doi:10.1371/journal.pgen.1011503)
Supplement: S3 Table — The table summarizes the number of discoveries for each of the six disease traits (rows) in the reduced sample TWAS fine-mapping analysis of UK Biobank. A GWAS risk region (1st column) is defined as an LD block that harbors at least one genome-wide significant SNP (p-value < 5 × 10^−8^). A significant TWAS gene (2nd column) is defined as a gene with a marginal TWAS p-value < 0.05/14,388. A TWAS risk region (3rd column) is defined as an LD block that harbors at least one marginal TWAS significant gene. A risk region with GWAS or TWAS signals (4th column) is defined as an LD block that harbors at least one genome-wide significant SNP or significant TWAS gene. The last three columns list the number of genes discovered by each of the three methods. The number in the bracket is the number of identified genes that are located in a risk region with GWAS or TWAS signals. We used an estimated FDR threshold of 0.05 to declare significance for all methods in the fine-mapping analysis. (DOCX) [file pgen.1011503.s003.docx]

S3 Table. Summary results of TWAS fine-mapping in UK Biobank (down-sampled)

| Trait | GWAS risk regions | Significant  TWAS genes | TWAS risk regions | Risk regions with GWAS or TWAS signals | FABIO | FOCUS | FOGS |
| --- | --- | --- | --- | --- | --- | --- | --- |
| AS | 8 | 1 | 1 | 9 | 9 (5) | 21 (2) | 62 (3) |
| BRCA | 5 | 0 | 0 | 5 | 7 (1) | 5 (0) | 45 (1) |
| GO | 10 | 11 | 8 | 16 | 21 (8) | 22 (4) | 79 (6) |
| HT | 59 | 20 | 17 | 66 | 141 (44) | 76 (22) | 143 (27) |
| PRCA | 4 | 0 | 0 | 4 | 6 (3) | 6 (1) | 42 (0) |
| RA | 4 | 5 | 3 | 6 | 12 (5) | 9 (3) | 38 (3) |
