# Supplementary figures and images for: FABIO: TWAS fine-mapping to prioritize causal genes for binary traits
Source: PLoS Genet. 2024 Dec 2;20(12):e1011503. doi: 10.1371/journal.pgen.1011503 (PMC11649093; doi:10.1371/journal.pgen.1011503)

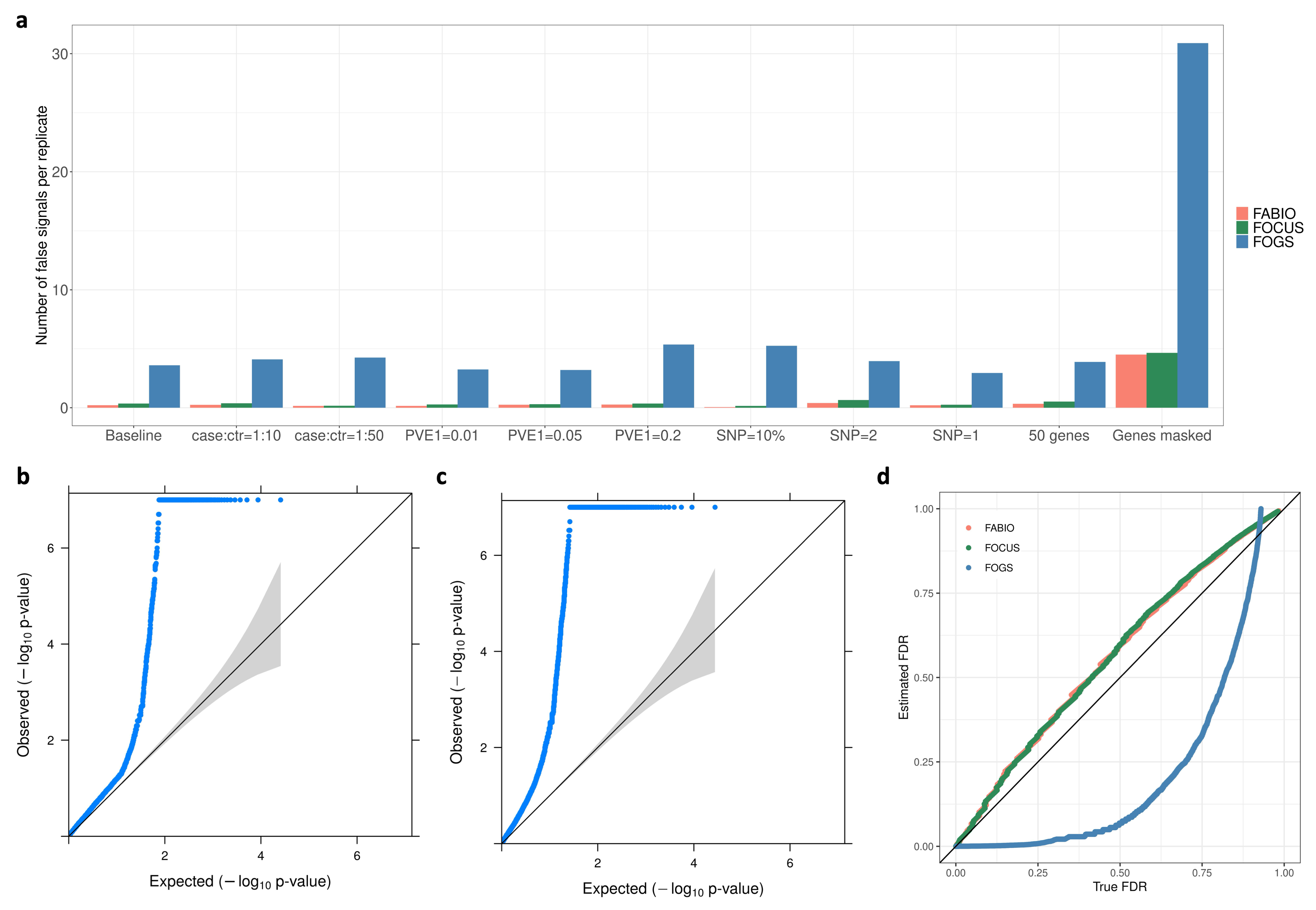

Supplement: S1 Fig — (a) Number of false signal genes per simulation replicate under different complete null settings (the first 10 settings) and the masked gene null setting (the last setting). (b) Quantile-quantile plot of -log10 p-values for testing the non-causal genes using FOGS under the complete null simulation setting. (c) Quantile-quantile plot of -log10 p-values for testing the non-causal genes using FOGS under the masked null simulation setting. (d) Comparisons among different methods for the estimate of false discovery rate (FDR) under the alternative baseline simulation setting. Compared methods include FABIO (salmon pink), FOCUS (green) and FOGS (blue). The estimated FDR (y-axis) is plotted against the true FDR (x-axis). (TIF) [file pgen.1011503.s004.tif]

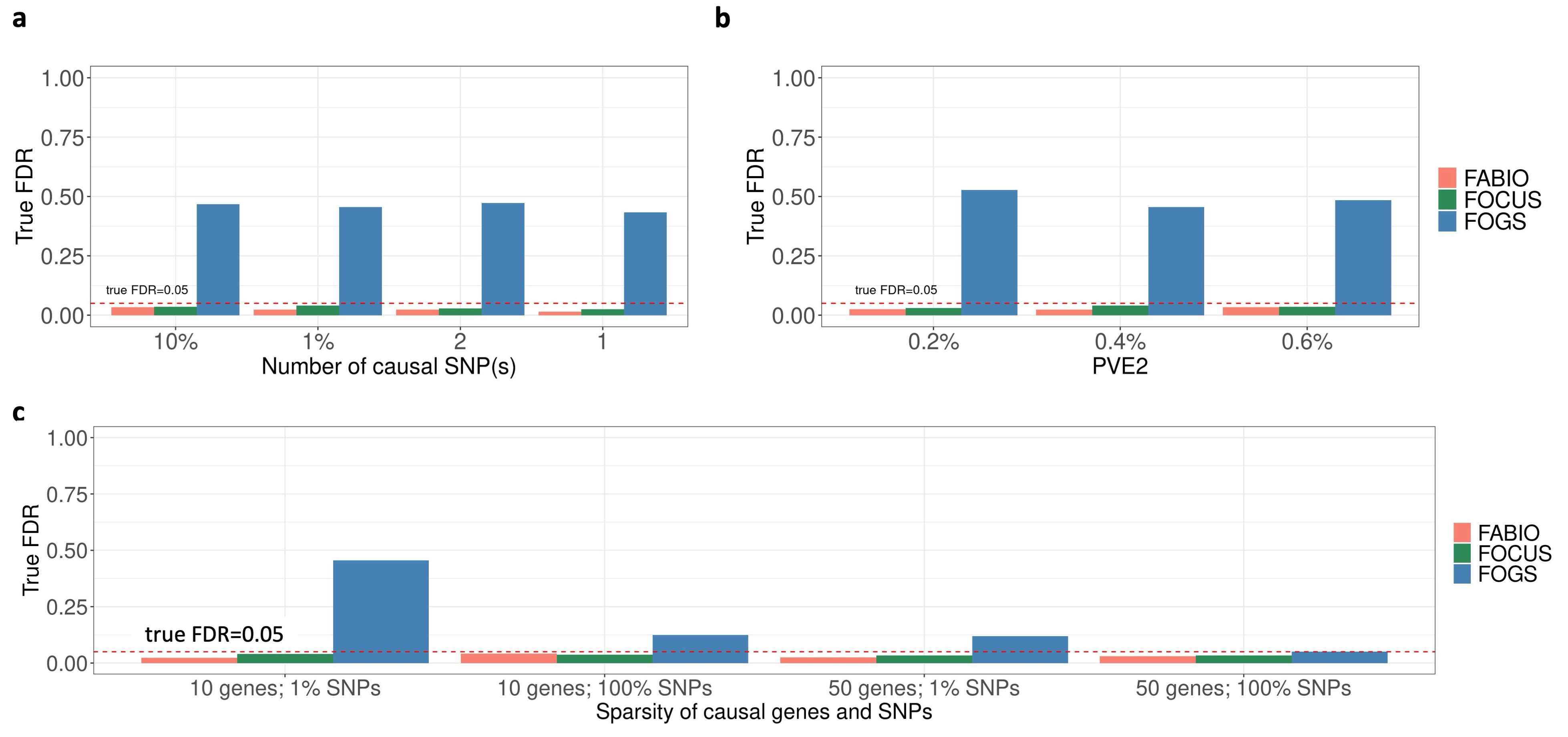

Supplement: S2 Fig — The red dashed line indicates a true FDR of 0.05. (a) Under different numbers of causal SNP(s). (b) Under different percentages of PVE2. (c) Under different sparsity of causal genes and SNPs. (TIF) [file pgen.1011503.s005.tif]

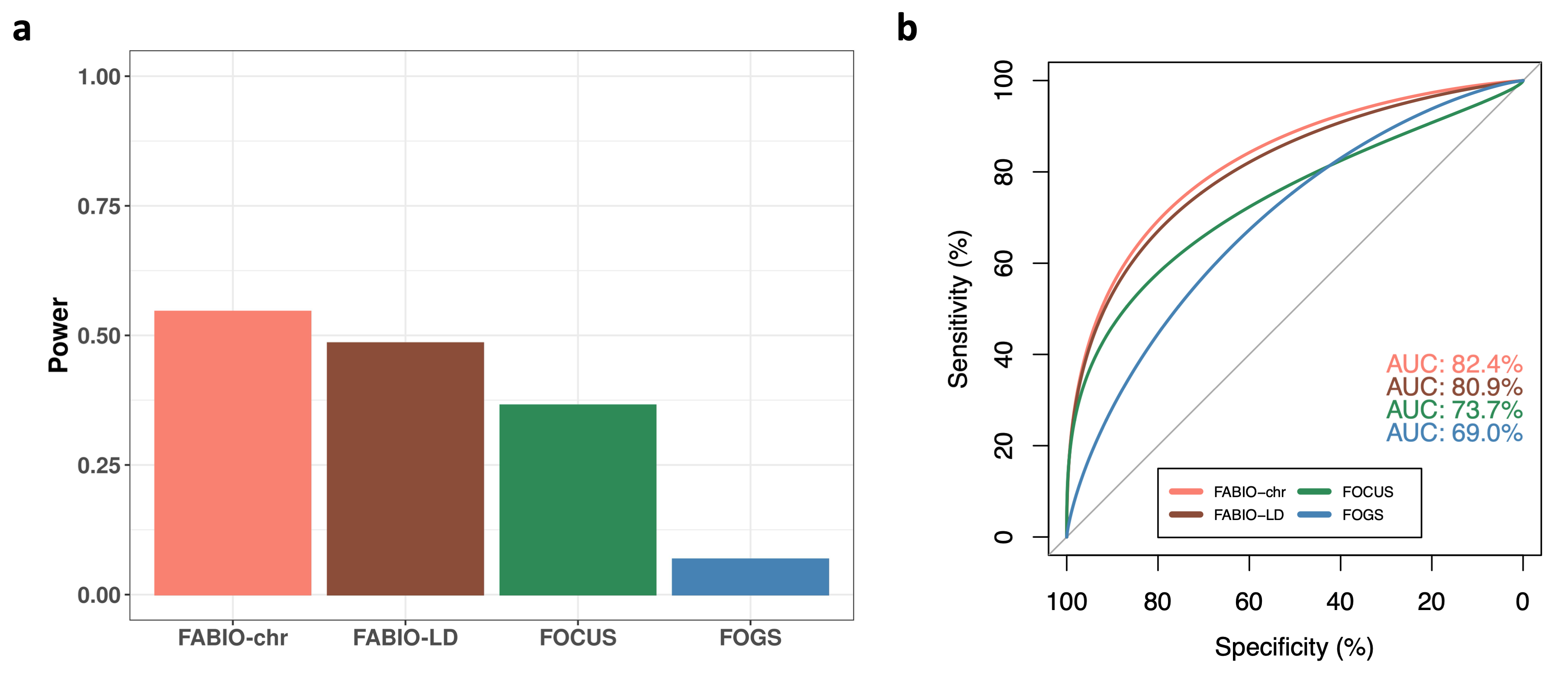

Supplement: S3 Fig — (a) Power comparison for different methods based on a true false discovery rate (FDR) of 0.05. FABIO was applied to either analyze all LD blocks jointly (FABIO-chr) or one LD block at a time (FABIO-LD). FOCUS and FOGS were only applied to analyze one LD block at a time. (b) ROC curves of different methods with AUCs recorded in the plot. (TIF) [file pgen.1011503.s006.tif]

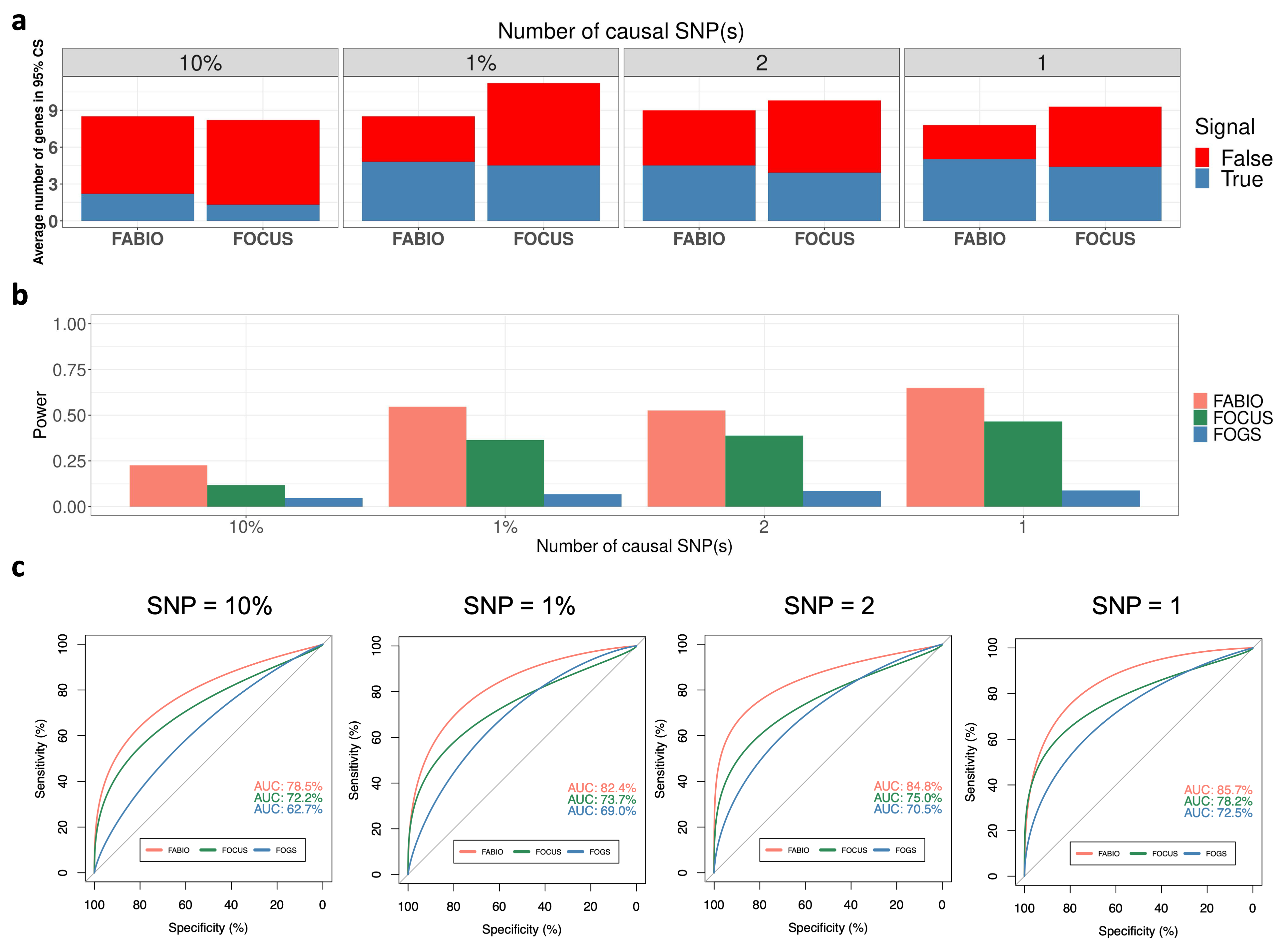

Supplement: S4 Fig — (a) Average number of genes in 95% credible set (CS) defined by FABIO or FOCUS and the number of true signal genes in 95% CS. (b) Power comparison for different methods based on a true false discovery rate (FDR) of 0.05. (c) ROC curves of different methods with AUCs recorded. (TIF) [file pgen.1011503.s007.tif]

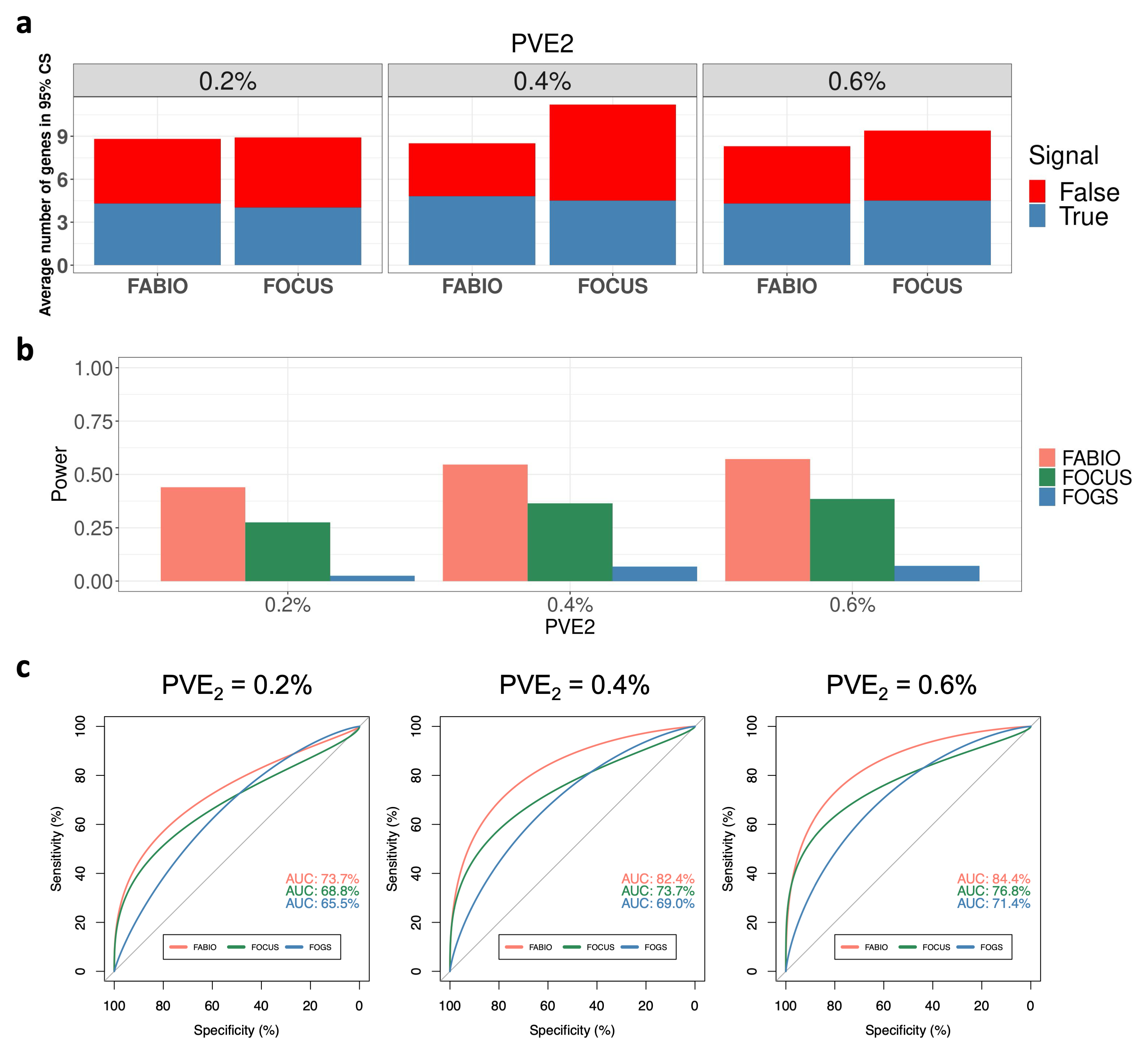

Supplement: S5 Fig — (a) Average number of genes in 95% credible set (CS) defined by FABIO or FOCUS and the number of true signal genes in 95% CS. (b) Power comparison for different methods based on a true false discovery rate (FDR) of 0.05. (c) ROC curves of different methods with AUCs recorded. (TIF) [file pgen.1011503.s008.tif]

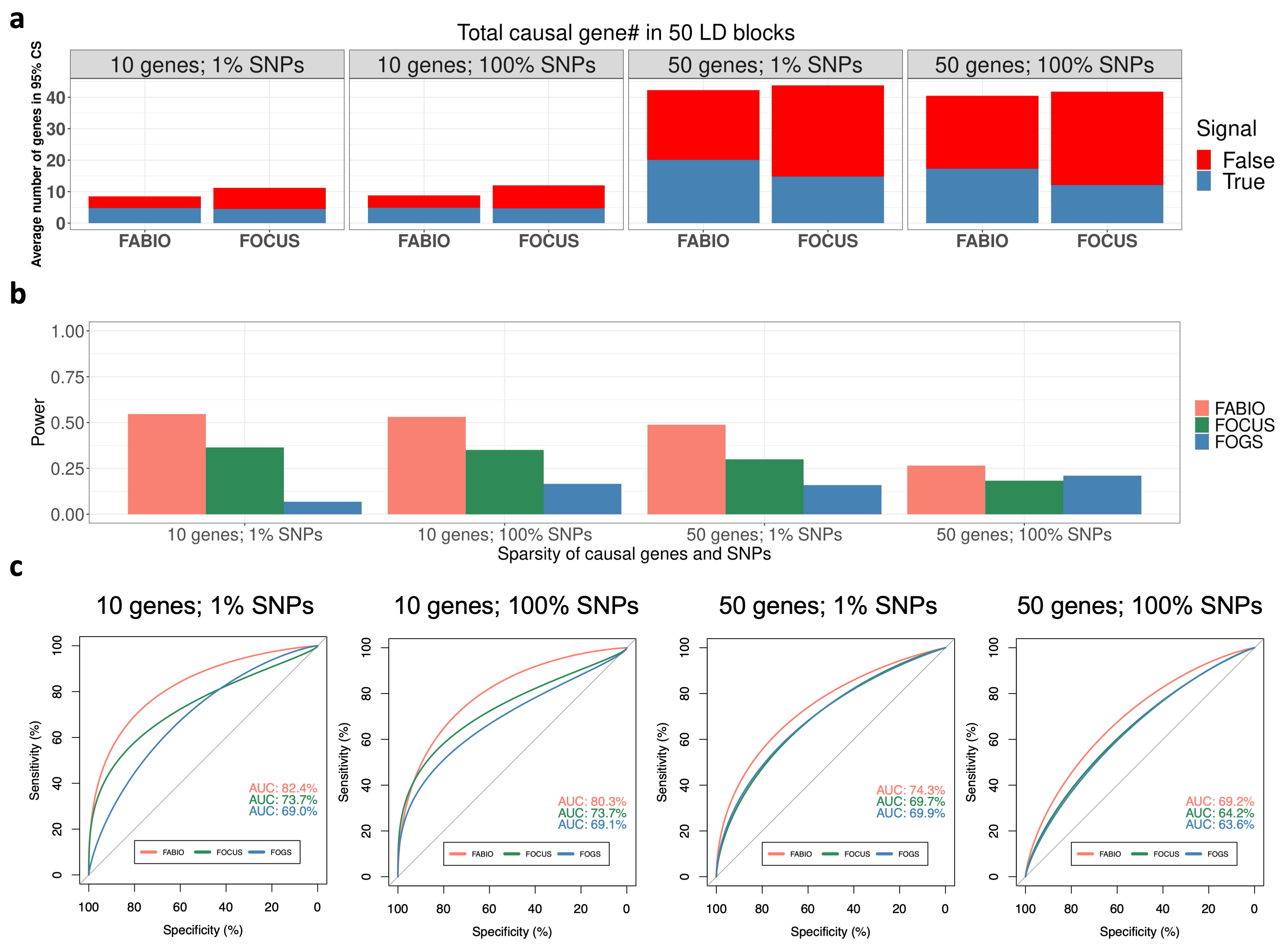

Supplement: S6 Fig — (a) Average number of genes in 95% credible set (CS) defined by FABIO or FOCUS and the number of true signal genes in 95% CS. (b) Power comparison for different methods based on a true false discovery rate (FDR) of 0.05. (c) ROC curves of different methods with AUCs recorded. (TIF) [file pgen.1011503.s009.tif]

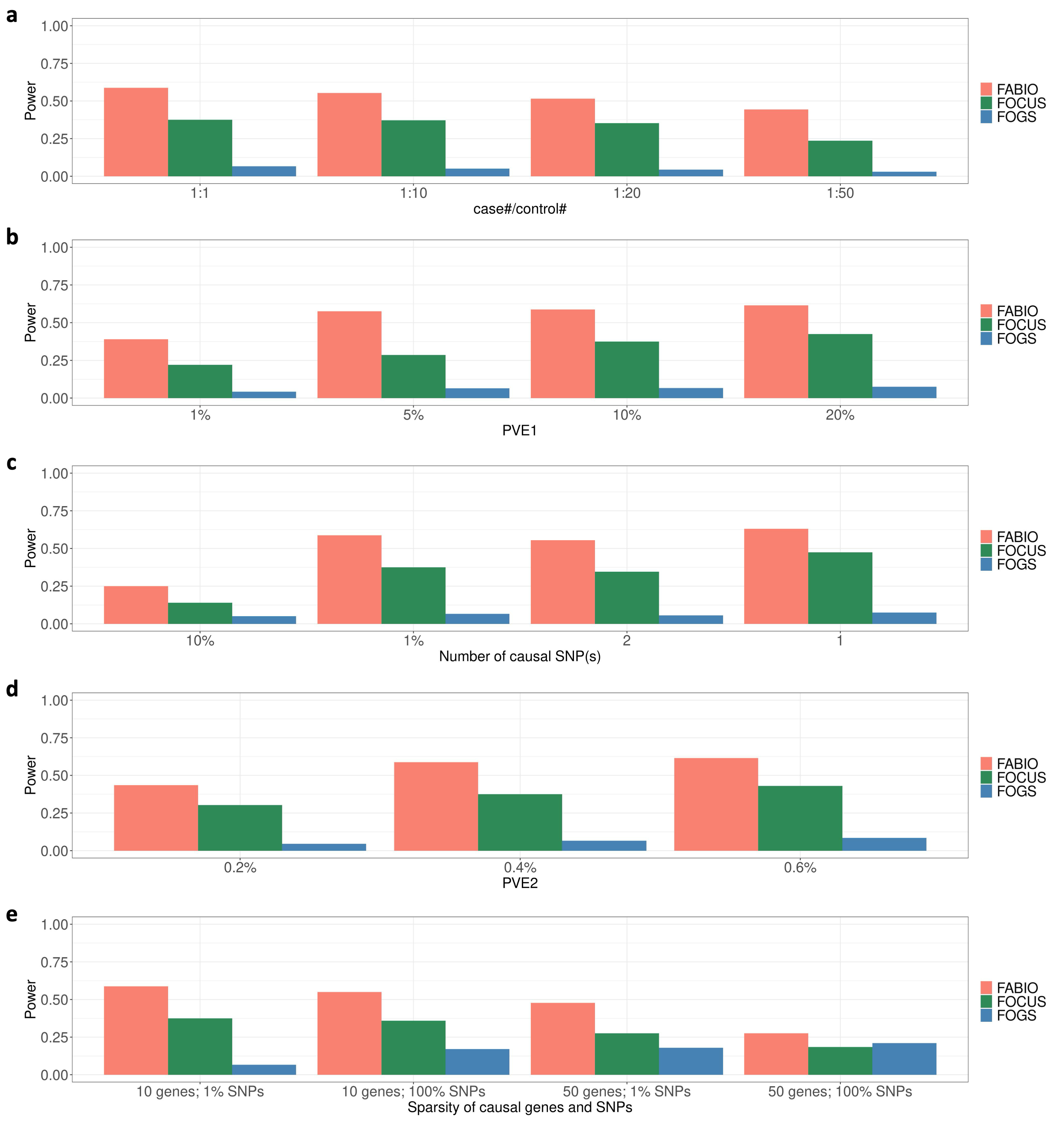

Supplement: S7 Fig — (a) under different case/control ratios. (b) under different PVE1. (c) under different numbers of causal SNP(s). (d) under different PVE2. (e) under different sparsity of causal genes and SNPs. (TIF) [file pgen.1011503.s010.tif]

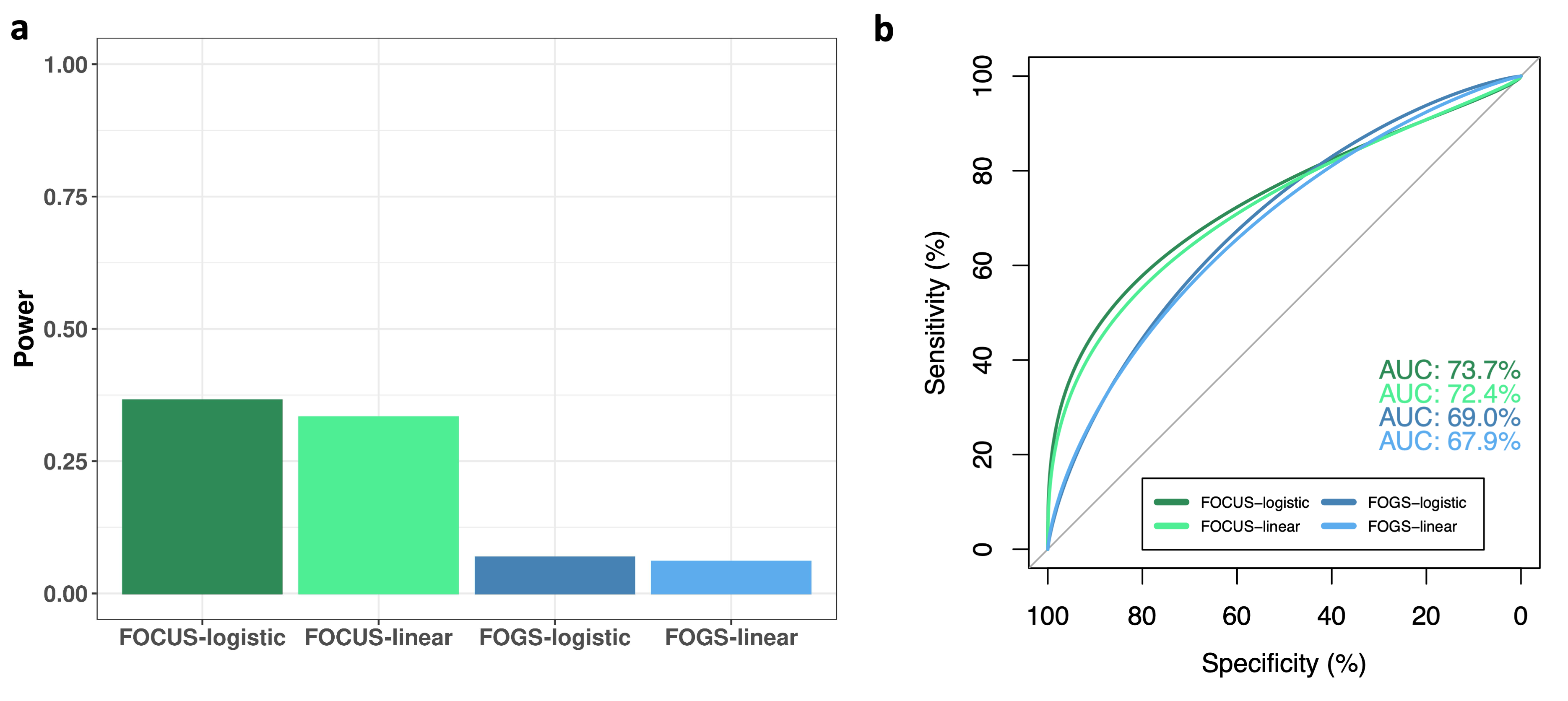

Supplement: S8 Fig — (a) Power comparison based on a true false discovery rate (FDR) threshold of 0.05. (b) ROC curves with AUCs recorded. (TIF) [file pgen.1011503.s011.tif]

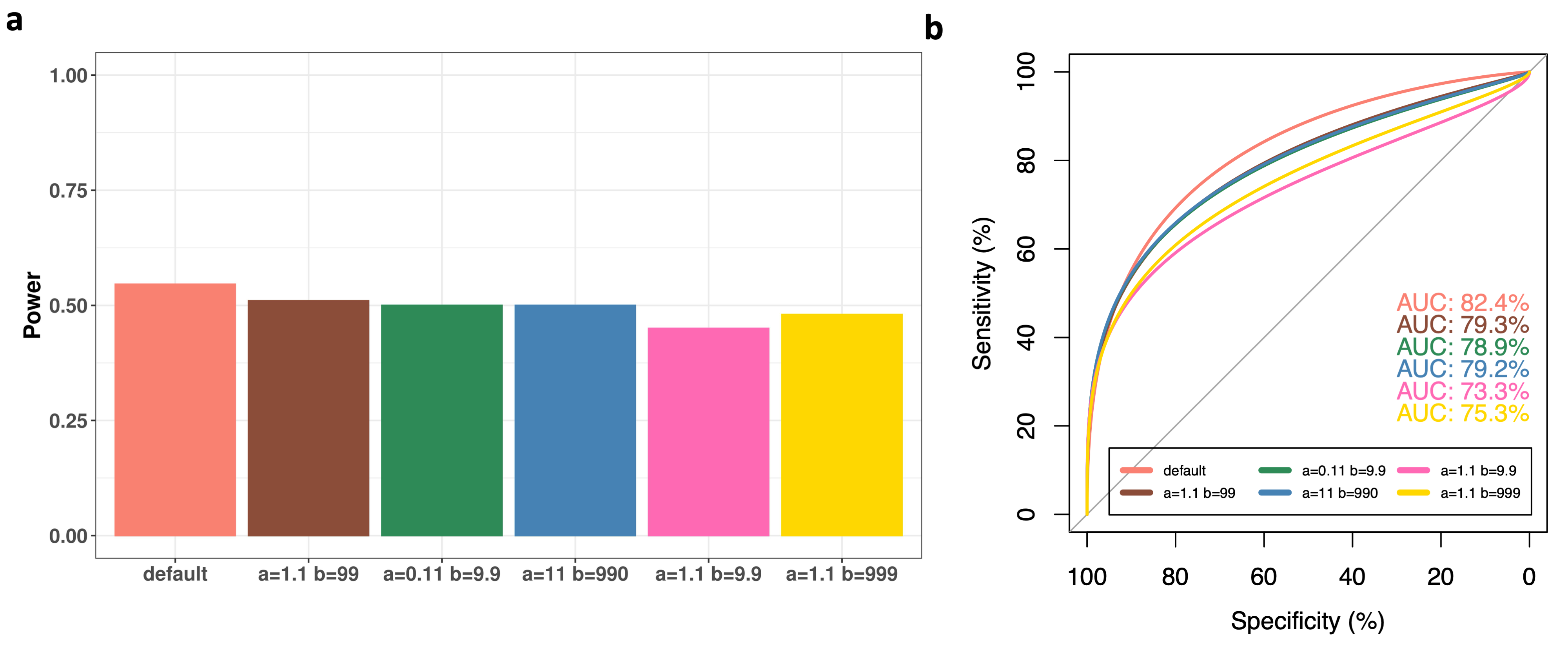

Supplement: S9 Fig — (a) Power comparison based on a true false discovery rate (FDR) threshold of 0.05. (b) ROC curves with AUCs recorded. (TIF) [file pgen.1011503.s012.tif]

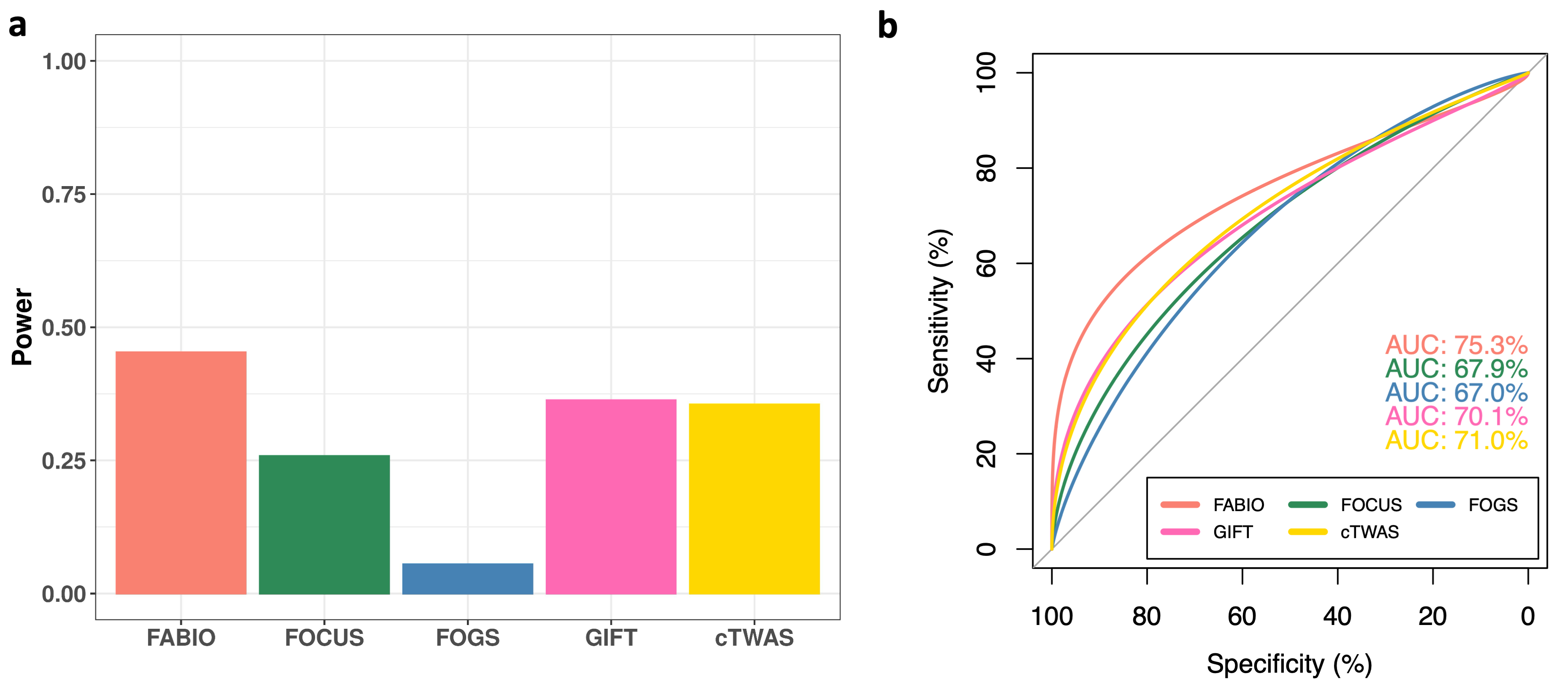

Supplement: S10 Fig — (a) Power comparison based on a true false discovery rate (FDR) threshold of 0.05. (b) ROC curves with AUCs recorded. (TIF) [file pgen.1011503.s013.tif]

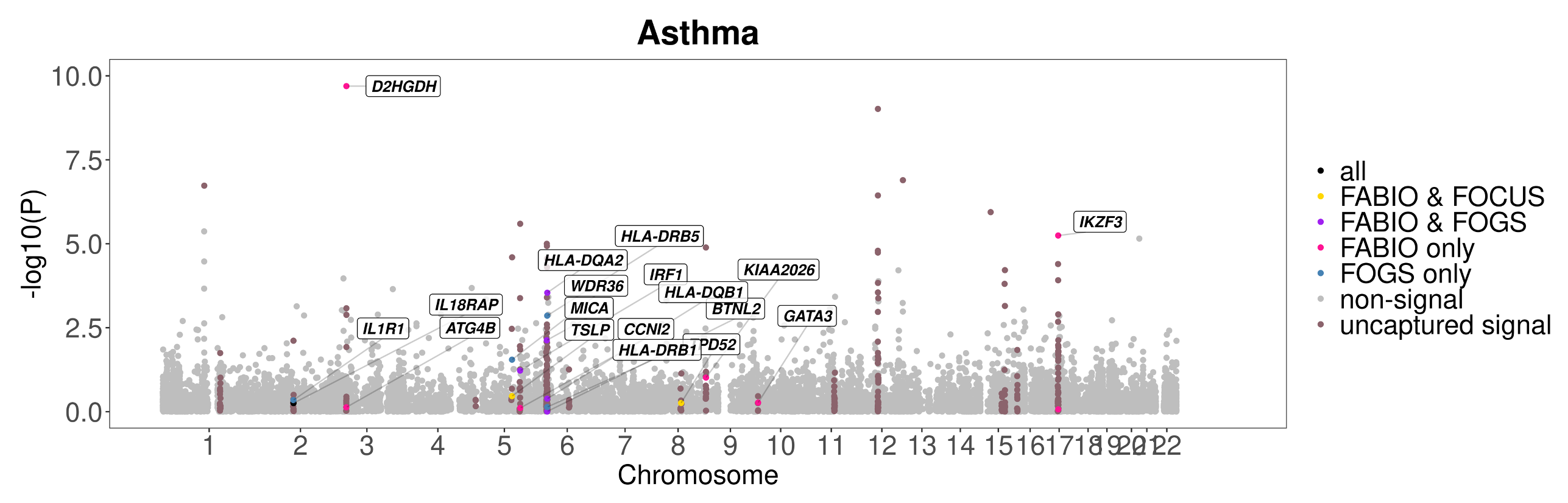

Supplement: S11 Fig — Genes identified in TWAS fine-mapping analysis are labeled in black boxes. Each gene is represented as a dot with x-axis indicating its genomic location and y-axis indicating its -log10 of p-value in the marginal TWAS association test. The dot of each gene is then colored based on different categories: (1) only identified by FABIO; (2) only identified by FOCUS; (3) only identified by FOGS; (4) identified by both FABIO and FOCUS; (5) identified by both FABIO and FOGS; (6) identified by both FOCUS and FOGS; (7) identified by all three methods; (8) located in a known risk region but missed by all three methods (uncaptured signal); (9) a gene shown no significance in both TWAS and GWAS analyses (non-signal). (TIF) [file pgen.1011503.s014.tif]

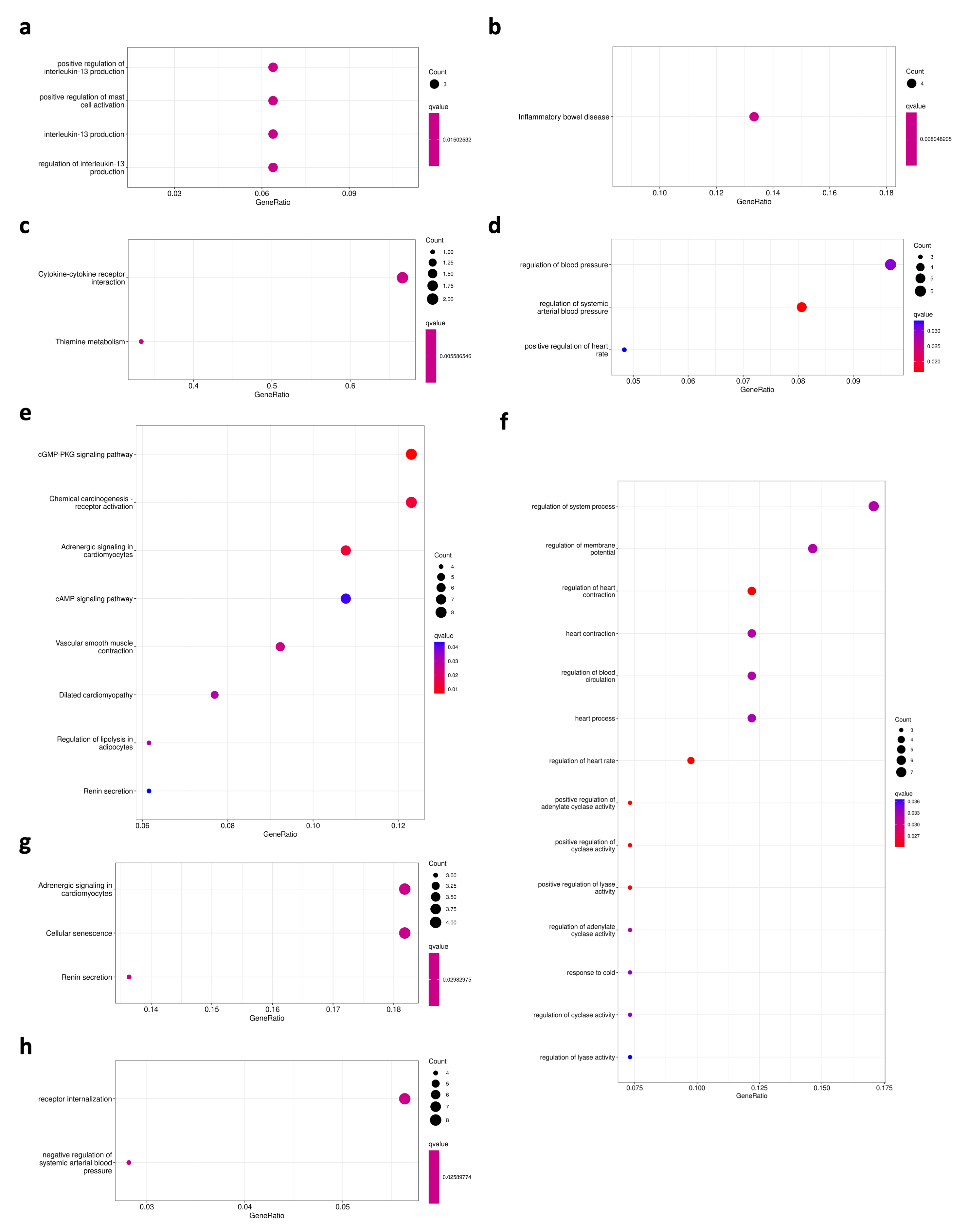

Supplement: S12 Fig — Dot plots show the significant terms of identified genes identified by different fine-mapping methods, with color gradients representing statistical significance based on q-value. (a) Gene Ontology enriched pathways for asthma using significant genes identified by FABIO. (b) KEGG enriched pathways for asthma using significant genes identified by FABIO. (c) KEGG enriched pathways for asthma using significant genes identified by FOCUS. (d) Gene Ontology enriched pathways for hypertension using significant genes identified by FABIO. (e) KEGG enriched pathways for hypertension using significant genes identified by FABIO. (f) Gene Ontology enriched pathways for hypertension using significant genes identified by FOCUS. (g) KEGG enriched pathways for hypertension using significant genes identified by FOCUS. (h) Gene Ontology enriched pathways for hypertension using significant genes identified by FOGS. (TIF) [file pgen.1011503.s015.tif]

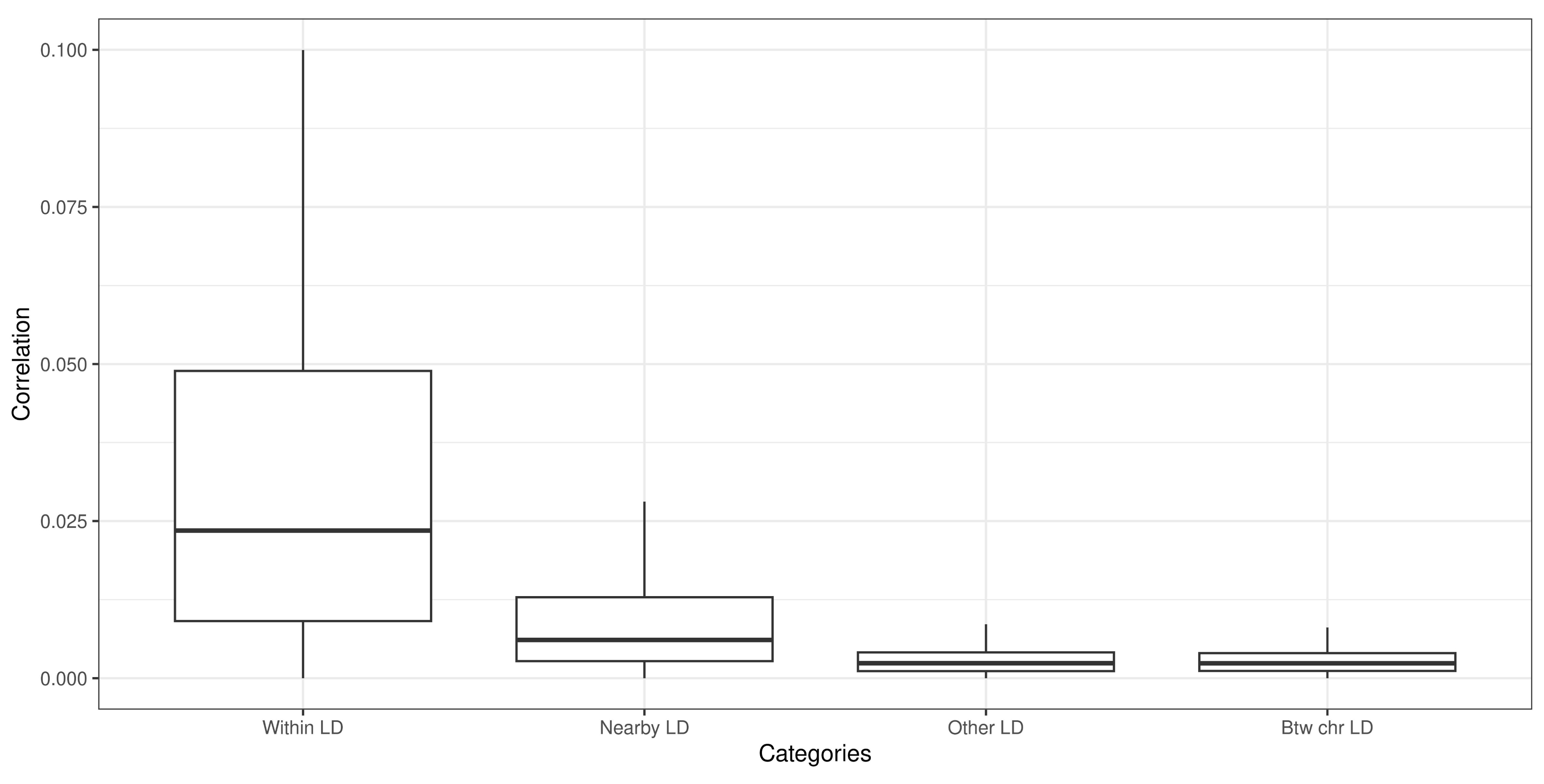

Supplement: S13 Fig — (1) Within LD: within each LD block, (2) Nearby LD: between two adjacent LD blocks, (3) Other LD: between two distant LD blocks, and (4) Btw chr LD: between one LD block on chromosome 1 and one on chromosome 2. (TIF) [file pgen.1011503.s016.tif]
