# Supplementary material for: FABIO: TWAS fine-mapping to prioritize causal genes for binary traits
Source: PLoS Genet. 2024 Dec 2;20(12):e1011503. doi: 10.1371/journal.pgen.1011503 (PMC11649093; doi:10.1371/journal.pgen.1011503)
Supplement: S1 Text — (DOCX) [file pgen.1011503.s017.docx]

**Prior specification of** $\boldsymbol{\pi}$ **and** $\boldsymbol{\sigma}^{\boldsymbol{2}}$

Our method involves two hyper parameters, $\pi$ and $\sigma^{2}$: $\pi$ controls the proportion of non-zero $\boldsymbol{\alpha}$ values in equation [3] of the main text; and $\sigma^{2}$ controls the expected magnitude of the non-zero $\boldsymbol{\alpha}$ values. Following [1], we first place a uniform prior on log $\pi$:

$\log\left( \pi\right)\sim U\left( \log\left( 1/p \right),\log\left( 1 \right) \right) .$ [9]

A common alternative is a uniform distribution directly on $\pi$ ^1^, while it would place large weights on large numbers of variables having non-zero effect in the model than a uniform prior on $\log\left( \pi\right)$. For example, it would assume a 50% prior probability to the event that more than half of the genes have non-zero effects, and a 90% prior probability to the event that more than 10% of the genes have non-zero effects. However, only a small proportion of genes are likely to contribute to the phenotype as we expected in practice, and the uniform prior on log $\pi$ is thus more suitable in this case. We also provide an alternative option of a beta prior distribution on $\pi: \pi\sim beta(a,b)$, where users can choose the values of *a* and *b* if they have prior knowledge to determine the prior expectation and the variance of $\pi$.

Following [1], we then re-parameterize the above model in terms of more interpretable quantities. Specifically, we define PVE as the total proportion of variance in latent variables $\boldsymbol{z}$ in equation [3] explained by the sparse effects of causal genes, and PVE can be represented as a function of $\hat{\mathbf{G}}$ and $\boldsymbol{\alpha}$:

$\mathrm{PVE}\left( \hat{\mathbf{G}}\boldsymbol{,\alpha} \right): =\frac{V(\hat{\mathbf{G}}\boldsymbol{\alpha})}{V\left( \hat{\mathbf{G}}\boldsymbol{\alpha} \right)+1} ,$ [10]

where the function $V\left( x \right)$ is defined as:

$V\left( x \right): =\frac{1}{n}\sum_{i=1}^{n} {(x_{i}-\bar{x})}^{2} .$ [11]

These definitions ensure that PVE will lie in the interval $\left[ 0,1 \right]$. PVE reflects how well we could predict the latent variable $\boldsymbol{z}$ in our model equation [3] from the GReX if we knew the optimal $\boldsymbol{\alpha}$.

Since PVE is a function of $\boldsymbol{\alpha}$, whose prior distribution depends on parameters $\pi$ and $\sigma^{2}$, the prior distribution for PVE will then depend on the priors assigned to these parameters. To simplify our model, we further aim to choose a prior for $\boldsymbol{\alpha}$ to induce the prior for PVE to be roughly uniform on 0 and 1. Following [2], we introduce *h* as an approximation (a ratio of expectations rather than an expectation of ratios) to the expectation of PVE:

$h : =\frac{p\pi s\sigma^{2}}{p\pi s\sigma^{2}+1} ,$ [12]

where $p$, $\pi$ and $\sigma^{2}$ are defined the same as before, $s$ is the average variance of expression level across genes: $s=\frac{1}{np}\sum_{i=1}^{p} \sum_{j=1}^{n} g_{ij}^{2}$, $g_{ij}$ is the $ij$th element of the matrix $\hat{\mathbf{G}}$. Here in equation [12], $p\pi$ is the expected number of causal genes in the model, and $\sigma^{2}$ is the expected effect size variance of each causal gene, and $s$ is the average variance of genes’ predicted expression level. Therefore, the whole term $p\pi s\sigma^{2}$ represents the expected variance contributed by the sparse gene effects. Through re-parameterizing the model in terms of ($h$, $\pi$) instead of ($\sigma^{2}$, $\pi$), now we can specify the uniform prior distribution for $h$, which is independent of $\pi$:

$h \sim U\left( 0,1 \right) ,$ [13]

Since $h$ approximates PVE, this prior distribution will lead to a reasonable uniform prior distribution for PVE. However, we only treat $h$ as the approximation to PVE for prior specification, we use PVE’s definition in equation [10] when estimating it directly from the real data.

**Posterior sampling**

Our goal is to obtain the posterior inclusion probability (PIP) of $\boldsymbol{\alpha}$, which represents the evidence for gene-trait association. To do so, we introduce a vector of binary indicators $\boldsymbol{\gamma}=\left( \gamma_{1},\cdots,\gamma_{p} \right)\in{\{0,1\}}^{p}$ to facilitate computation [3], where each $\gamma_{i}$ indicates whether the corresponding $\alpha_{i}$ is non-zero. Our point-normal priors for $\boldsymbol{\alpha}$ can then be written as:

$\gamma_{i} \sim\mathrm{Bernoulli}\left( \pi\right),$ [14]

$\boldsymbol{\alpha}_{\boldsymbol{\gamma}} \sim\mathrm{MVN}_{\left| \boldsymbol{\gamma} \right|}\left( 0,\sigma^{2}\mathbf{I}_{\left| \boldsymbol{\gamma} \right|} \right),$ [15]

$\boldsymbol{\alpha}_{\boldsymbol{-\gamma}} \sim\delta_{0},$ [16]

where $\boldsymbol{\alpha}_{\boldsymbol{\gamma}}$ denotes the sub-vector of $\boldsymbol{\alpha}$ corresponding to the entries $\{i:\gamma_{i}=1\}$; $\boldsymbol{\alpha}_{\boldsymbol{-\gamma}}$ denotes the sub-vector of $\boldsymbol{\alpha}$ corresponding to the other entries $\{i:\gamma_{i}=0\}$; and $|\boldsymbol{\gamma|}$ denotes the number of non-zero entries in $\boldsymbol{\gamma}$. We use MCMC to obtain posterior samples of ($h, \pi, \boldsymbol{\gamma}$) on the product space $\left( 0,1 \right)\times\left( 0,1 \right)\times\left\{ 0,1 \right\}^{p}$, which is given by:

$P\left( h,\pi,\boldsymbol{\gamma} | \boldsymbol{y} \right)\propto P\left( \boldsymbol{y} | h, \pi, \boldsymbol{\gamma} \right)P\left( h \right)P\left( \boldsymbol{\gamma} | \pi\right)P\left( \pi\right) ,$ [17]

We also sample the posteriors of the latent variables $\boldsymbol{z}$ using the conditional distribution $P(\boldsymbol{z}|\boldsymbol{y},\boldsymbol{\gamma,}\boldsymbol{\alpha})$:

$z_{i}|y_{i}=1, \boldsymbol{\alpha}\boldsymbol{\sim}N\left( \mu+{{\hat{\boldsymbol{g}}}_{\gamma_{i}}}^{T}\boldsymbol{\alpha,}1 \right) left truncated at 0 ,$ [18]

$z_{i}|y_{i}=0, \boldsymbol{\alpha\sim}N\left( \mu+{{\hat{\boldsymbol{g}}}_{\gamma_{i}}}^{T}\boldsymbol{\alpha,}1 \right) right truncated at 0 .$ [19]

Conditional on the latent variables $\boldsymbol{z}$, the posterior sampling for the parameters ($h,\pi,\boldsymbol{\gamma}$) will be based on the marginal likelihood $P\left( h,\pi,\boldsymbol{\gamma} | \boldsymbol{z} \right)$. After integrating $\boldsymbol{\alpha}$ out, the marginal likelihood can be represented as:

$P\left( \boldsymbol{z} | h,\pi,\boldsymbol{\gamma} \right)\propto\left| \sigma^{-2}\boldsymbol{\Omega} \right|^{\frac{1}{2}}e^{-\frac{1}{2}\left( \boldsymbol{z-}\boldsymbol{1}_{n}\bar{z} \right)^{T}\mathbf{P}\left( \boldsymbol{z-}\boldsymbol{1}_{n}\bar{z} \right)} .$ [20]

where $\boldsymbol{\Omega}\boldsymbol{=}{\boldsymbol{(}{\hat{\mathbf{G}}}_{\boldsymbol{\gamma}}^{T}{\hat{\mathbf{G}}}_{\boldsymbol{\gamma}}\boldsymbol{+}\sigma^{-2}\mathbf{I}_{\left| \boldsymbol{\gamma} \right|}\boldsymbol{)}}^{-1}$, ${\hat{\mathbf{G}}}_{\boldsymbol{\gamma}}$ is an *n* × $|\boldsymbol{\gamma|}$ sub-matrix of $\hat{\mathbf{G}}$ that only contains predicted expression level of genes with non-zero effects, $\bar{z}=\frac{1}{n}\sum_{i=1}^{n} z_{i}$ is the sample mean of $\boldsymbol{z}$, and $\mathbf{P}\mathbf{=}\mathbf{I}_{n}-{\hat{\mathbf{G}}}_{\boldsymbol{\gamma}}\boldsymbol{\Omega}{\hat{\mathbf{G}}}_{\boldsymbol{\gamma}}^{T}$.

We use a standard Metropolis-Hastings algorithm to draw posterior samples of ($h,\pi,\boldsymbol{\gamma}$) from the above marginal distribution, starting with a rank-based proposal distribution for $\boldsymbol{\gamma}$ [1]. The advantage of this rank-based distribution is to focus more on examining genes with stronger marginal associations. To be more detailed, we first calculate the p-value for each gene using the standard LMM algorithm [4], and then rank genes based on their p values from small to large. To put more weights on genes that have higher rank in the single-gene tests, we propose with a mixture distribution $Q_{p}=0.3U_{p}+0.7G_{p}$ following [2]. Here, $U_{p}$ is a uniform distribution on 1, 2, …, $p$ and $G_{p}$ is a geometric distribution truncated to 1, 2, …, $p$ with its parameter chosen to give a mean of 2000. We denote $\boldsymbol{\gamma}^{+}=\{i:\gamma_{i}=1\}$ and propose the new $\boldsymbol{\gamma}$ by randomly choosing from one of the following options:

Option 1 (with probability 0.4): generate a number $r$ from $Q_{p}$ until the covariate with rank $r$ is not in $\boldsymbol{\gamma}^{+}$, then add this covariate to $\boldsymbol{\gamma}^{+}$;

Option 2 (with probability 0.4): randomly (uniformly) select a covariate in $\boldsymbol{\gamma}^{+}$ and remove it from $\boldsymbol{\gamma}^{+}$;

Option 3 (with probability 0.2): select two covariates by the above two options and then switch their indicator values.

We then apply the random walk proposals based on uniform distributions for $h$ and $\log\left( \pi\right)$: we update $h$ by adding a random variable from $U(-0.1,0.1)$ to the current value, and update $\log\left( \pi\right)$ by adding a random variable from $U(-0.05,0.05)$ to the current value. New values that beyond the range from 0 to 1 will be reflected back. To improve the theoretical MCMC convergence, we also apply a “small world proposal” [5] in addition to the local proposals illustrated above. In the “small world proposal”, we compound multiple local moves instead of a single move to induce longer-range proposals on the proposal distributions. The number of compounded local moves is uniformly drawn from a uniform distribution ranging from 1 to 20.

After obtaining the posterior samples of $\boldsymbol{z}$ and the parameters ($h,\pi,\boldsymbol{\gamma}$), we sample the posterior of $\boldsymbol{\alpha}$ using its conditional distribution:

$\boldsymbol{\alpha}_{\boldsymbol{\gamma}}|\boldsymbol{z,}h,\pi,\boldsymbol{\gamma}\sim\mathrm{MVN}_{\left| \boldsymbol{\gamma} \right|}\left( \boldsymbol{\Omega}{\hat{\mathbf{G}}}_{\boldsymbol{\gamma}}^{T}\boldsymbol{z},\boldsymbol{\Omega} \right) ,$ [21]

$\boldsymbol{\alpha}_{\boldsymbol{-\gamma}}|\boldsymbol{z,}h,\pi,\boldsymbol{\gamma} \sim\delta_{0} .$ [22]

At the end, we sample $\mu$ in equation [3] based on the conditional distribution:

$\mu|\boldsymbol{z,\alpha,\gamma\sim}N\left( \frac{1}{n}\boldsymbol{1}_{\boldsymbol{n}}^{T}\left( \boldsymbol{z-}{\hat{\mathbf{G}}}_{\boldsymbol{\gamma}}\boldsymbol{\alpha}_{\boldsymbol{\gamma}} \right)\boldsymbol{,}\frac{1}{n} \right)\boldsymbol{.}$ [23]

**Model specifications for FOCUS**

FOCUS examines genomic loci one at a time. For a particular locus that contains p SNPs for 𝑚 genes, FOCUS relies on a multiple linear regression model to model a quantitative trait $\mathbf{y}$ as a linear combination of gene expression levels in the form of $\mathbf{y}=\hat{\mathbf{G}}\boldsymbol{\alpha+}\lambda\boldsymbol{\beta}+\boldsymbol{\varepsilon}$, where $\mathbf{y}$ is a centered phenotype vector for n individuals, $\hat{\mathbf{G}}$ is an n × m predicted genetically regulated expression (GReX) matrix, 𝛂 is an m × 1 vector of corresponding gene effects, $\boldsymbol{\beta}$ is an n × 1 vector of covariant, $\lambda$ is a scalar of the corresponding covariant effect, and $\boldsymbol{\varepsilon}$ is a random noise term with E[$\boldsymbol{\varepsilon}$] = 𝟎 and V[$\boldsymbol{\varepsilon}$] = $\mathbf{I}_{n}\sigma_{e}^{2}$. Same as FABIO, FOCUS relies on the predicted GReX in the form of $\hat{\mathbf{G}}\boldsymbol{=X\Omega}$, where $\boldsymbol{\Omega}$ is a p × m weight matrix representing the SNP effects on gene expression estimated from a separate expression mapping study. Because of the linear modeling framework, the above model can also be translated into a model based on marginal GWAS summary statistics, in the form of the estimated SNP effects on gene expression, SNP LD matrix, and SNPs Z-scores from marginal GWAS analysis.

**Model specifications for FOGS**

FOGS examines genomic loci one at a time. For a particular locus with 𝑚 genes, FOGS further examines one gene at a time. For a gene of focus, FOGS first divides the cis-SNPs in the locus into two sets: a set of p SNPs with non-zero effects on the expression of the gene of focus and another set of q SNPs with non-zero effects on the expression of other genes in the locus. Afterwards, FOGS relies on a multiple linear regression model in the form of $\mathbf{y}=\boldsymbol{X\beta+V\alpha}+\boldsymbol{\varepsilon}$, where **y** is a centered phenotype vector for n individuals, **X** is a centered n × p genotype matrix at p SNPs with non-zero effects on expression of the target gene, **V** is a centered n × q genotype matrix at q SNPs with non-zero effects on expression of the remaining m-1 genes, and $\boldsymbol{\beta}$ is a p × 1 vector of the SNP effects on the target gene expression while$\boldsymbol{\alpha}$ is a q × 1 vector of the SNP effects on the remaining genes. FOGS tests the causal effect between the phenotype and the target gene under the null hypothesis H_0_: $\boldsymbol{\beta}=\mathbf{0}$. For the target gene with p SNPs, FOGS further uses ridge regression to estimate the p conditional Z scores for those SNPs by converting marginal effects obtained from GWAS summary statistics to joint effects. Afterwards, to aggregate Z scores of multiple SNPs from the same gene, FOGS applies a weighted adaptive sum of powered score (SPU) test with eQTL-derived weights, and generates the p-value for each gene as the evidence of causality. Since the underlying truth for the effect sizes and directions of SNPs is unknown, FOGS constructs a class of SPU tests using a positive integer $\gamma$ as the tuning parameter. As $\gamma$ increases, $SPU(\gamma)$ becomes more powered to detect signals where a sparse set of SNPs display effects on phenotype. FOGS further selects the optimal value of $\gamma$, where $SPU(\gamma)$ achieves the smallest p-value. FOGS uses $\gamma\in\left\{ 1,2,3,4,5,6 \right\}$ as the default setting; thus FOGS is expected to be more powerful when the true SNP effects on the trait are not sparse. Overall, FOGS uses marginal GWAS summary statistics, in the form of the estimated SNP effects on gene expression, SNP LD matrix, and SNPs Z-scores from marginal GWAS analysis.

**References**

1. Guan Y & Stephens M. Bayesian variable selection regression for genome-wide association studies and other large-scale problems. *The Annals of Applied Statistics* **5**, 1780-1815 (2011).

2. Zhou X, Carbonetto P, & Stephens M. Polygenic modeling with Bayesian sparse linear mixed models. *PLoS genetics* **9**, e1003264 (2013).

3. George EI & McCulloch RE. Variable selection via Gibbs sampling. *Journal of the American Statistical Association* **88**, 881-889 (1993).

4. Molenberghs G & Verbeke G. *Linear mixed models for longitudinal data*. (Springer, 1997).

5. Guan Y & Krone SM. Small-world MCMC and convergence to multi-modal distributions: From slow mixing to fast mixing. *The Annals of Applied Probability* **17**, 284-304 (2007).
