# Supplementary material for: FABIO: TWAS fine-mapping to prioritize causal genes for binary traits
Source: PLoS Genet. 2024 Dec 2;20(12):e1011503. doi: 10.1371/journal.pgen.1011503 (PMC11649093; doi:10.1371/journal.pgen.1011503)
Supplement: Attachment — Submitted filename: response_letter.docx [file pgen.1011503.s018.docx]

**Comments From Reviewer #1:**

In this manuscript, you present FABIO, a novel multivariate TWAS method aimed at identifying disease-associated genes. FABIO offers two key advantages over existing methods. First, it is specifically designed for binary traits using individual-level data. Second, it can simultaneously analyze all genes on a chromosome, rather than focusing on a single gene or multiple genes within a single cis-region, as is the case with current methods. I highly commend the methodological innovation of this work and anticipate that it will play a significant role in future TWAS analyses. However, there are some issues with the presentation of the manuscript, and I have a few questions that I hope the authors can address or clarify.

Thank you for your positive and constructive comments. Our detailed responses to each of your comment is listed below.

Treating binary traits as continuous in existing methods?

To my knowledge, existing methods such as FOCUS and FOGS, which are based on GWAS summary data, do not approximate binary variables as continuous ones. In fact, for binary traits, GWAS tests the marginal correlations between a variant and the liability score of this binary trait (i.e., the latent variable z_i in FABIO, equations 2 and 3). While there may be bias due to model misspecification (i.e., the generating model is a liability model (probit model) while the estimation model is a logistic model), this is not the same issue as the one claimed by the authors in the second paragraph of the introduction. The authors also state in their simulations that these existing methods are based on GWAS summary data, rather than performing a linear model on binary outcomes. As mentioned in the General Comment, I have no doubt about the methodological innovation of FABIO, but it does not address these specific issues with existing methods.

Thank you for your comments. We response to each of your comments here in detail below.

Regarding your first comment “GWAS tests the marginal correlations between a variant and the liability score of this binary trait”, we apologize for not adequately explaining standard GWAS marginal analysis, which may have led to this misunderstanding. In fact, standard marginal GWAS analysis typically relies on either linear regression, treating the binary trait as a continuous outcome, or logistic regression, which accounts for the binary nature of the trait, to compute the marginal GWAS summary statistics. We are not aware of any publicly available GWAS summary statistics derived from a liability threshold model, at least not from the majority of consortium studies, as fitting a liability model is computationally demanding, and inferring liability scores is not statistically straightforward. The only two GWAS marginal analysis methods we know of that use a liability threshold model to compute summary statistics are Weissbrod et al., 2015 and Hujoel et al., 2020. Both approaches rely on a mixed model framework and are computationally demanding for large scale GWAS. However, for the purposes of the discussion here, it does not matter which marginal GWAS model was used to generate these GWAS summary statistics. What is critical is distinguishing the model used by TWAS fine-mapping methods and the input GWAS summary statistics, which are generated by GWAS marginal analysis methods (as further discussed in the following paragraphs).

Regarding your second comment “While there may be bias due to model misspecification (i.e., the generating model is a liability model (probit model) while the estimation model is a logistic model), this is not the same issue as the one claimed by the authors in the second paragraph of the introduction.”, we believe this reflects a potential miscommunication stemming from two distinct issues. Specifically, in the second paragraph of the introduction, we were primarily discussing the modeling issue in the TWAS fine-mapping methods, FOCUS and FOGS. However, your comment appeared to focus on the issue related to the input GWAS summary statistics, which are generated by marginal GWAS methods (not TWAS methods) and then used as input to the TWAS fine-mapping methods.

In terms of the model, existing TWAS fine-mapping methods, FOCUS and FOGS, use a linear regression modeling framework rather than a logistic regression or a probit model. Consequently, while the data generating model for binary traits may be logistic or probit, the estimation model in FOCUS and FOGS remains linear. This is why we identified this as the first limitation of these two methods in the second paragraph of the introduction. Based on your comment, we have added detailed modeling specifications for FOCUS and FOGS in the Supplementary Text to clarify their linear modeling framework and assumptions (**sections “Model specifications for FOCUS” and “Model specifications for FOGS” on pages 5-7**).

In terms of GWAS summary statistics input, the modeling framework of FOCUS and FOGS suggests using marginal GWAS from linear regression. However, their software is not restricted to this and can directly use summary statistics output from any marginal GWAS analysis. This is justified given the high similarity between summary statistics generated from different marginal analysis methods. Indeed, linear regression for marginal analysis is an accurate approximation to the logistic regression when the SNP effect sizes are small, which is typically the case for most GWAS (Zhou et al., 2013). For example, in our simulations, the correlation of marginal GWAS z-scores between linear and logistic regressions across SNPs averaged 98.92% between logistic regression and linear regression. Therefore, the software of FOCUS and FOGS can work with marginal GWAS summary statistics derived from linear, logistic, or even probit regression models, although the latter is much less common as discussed earlier. Throughout this study, we have used summary statistics obtained from logistic regression as input for FOCUS and FOGS (as well as FABIO) in both simulations and real data applications.

Importantly, the type of GWAS marginal analysis used to generate these summary statistics does not alter the TWAS fine-mapping model employed by FOCUS, FOGS, or FABIO, nor does it significantly impact the final results. To demonstrate this, we conducted new analyses in the simulations where we used linear regression as the GWAS marginal analysis, treating binary traits as continuous outcomes to generate marginal GWAS summary statistics as input for FOCUS and FOGS. The comparison showed very similar or slightly reduced performance when using linear regression compared to using logistic regression: at a true FDR threshold of 0.05, the power for FOCUS was 33.3% and 36.5%, and for FOGS, 6.0% and 6.8%, for using linear and logistic regression GWAS summary statistics, respectively. These results are summarized in the updated **Figure S8**. Based on these new results, we have retained the use of logistic regression GWAS summary statistics as input for both FOCUS and FOGS.

Regarding your last comment “The authors also state in their simulations that these existing methods are based on GWAS summary data, rather than performing a linear model on binary outcomes.”, indeed, we can confirm that we previously used the marginal GWAS summary statistics obtained from logistic regression as input for FOCUS, FOGS, and FABIO in both simulations and real data applications. But hopefully this last comment has been fully addressed by the detailed explanations provided in responses to your second comment above.

Overall, your comments have made it clear that we previously did not explain the modeling and input issues separately and clearly. Following your comments, we have revised our manuscript to better distinguish between the modeling framework and the input GWAS summary statistics. These clarifications have been incorporated into the updated Introduction (**lines 94-99 on page 5**), Results (**lines 301-307 on pages 15-16**), and Supplementary Text (**sections “Model specifications for FOCUS” and “Model specifications for FOGS” on pages 5-7**).

Fairness of simulation for FOGS

I observed unusual results in Figures 2 and 3: FOGS has a very high type-I error (based on FDR) and extremely low power. After reading the authors' processing methods, I found that they used simulations to discover the potential type-I error of each method (2.6% for FABIO, 3.3% for FOCUS, 46.5% for FOGS) and then used these thresholds to calculate power. This is unfair to FOGS. After reading about FOGS, I discovered that it is not based on a sparsity assumption, and it is normal for its type-I error to be higher than that of FABIO and FOCUS, which utilize a sparsity assumption. The authors then use this very high type-I error as the threshold for determining power, which exaggerates the shortcomings of FOGS. In addition, the terminology "true FDR of 0.05" is somewhat misleading. I believe that the analysis of type-I error and power should be independent, and the threshold should be chosen using the same method (e.g., q-value < 0.05). The phrase "simulated threshold of 0.05 FDR" may be more appropriate and fair.

Thank you for your comments. First, let us clarify what we mean by "true FDR" and "estimated FDR" in the manuscript, which we hope will address the main concerns raised in your comment. Following this explanation, we will respond to each of your points individually.

In the previous manuscript, we calculated FDR in two different ways, corresponding to the terminologies “true FDR”, which was used in simulations where the ground truth is known, and “estimated FDR”, which was used in both simulations and real data applications where the ground truth is unknown. The purpose of doing either is to establish a common threshold for method comparison, as suggested in your comment.

Specifically, to compute the true FDR, in the simulations, we first divided 100 simulation replicates into five groups, with 20 replicates per group. In each group, we obtained the test statistics from each method for all genes across the 20 replicates, either in the form of p-values or PIPs. For each method in turn, we sorted the test statistics from the most significant (i.e. the smallest p-value or the largest PIP) to the least significant (i.e. the largest p-value or the smallest PIP). For each ordered test statistic in the sorted list, we counted the number of false positives detected above the threshold defined by this specific test statistic. The number of false positives, divided by the total number of genes called significant at that threshold, is FDR. We then determined threshold that corresponds to an FDR of 0.05, based on which we further calculated power. Power is calculated as the number of true positives above the threshold in the sorted list divided by the total number of causal genes. Afterwards, we obtained the average power across five groups to minimize stochasticity.

Because we do not have the ground truth, we cannot compute “true FDR” in the real data. Instead, we estimated FDR using different approaches: the Benjamini-Hochberg approach to estimate FDR using p-values from FOGS, and the local FDR approach (Efron 2007) to estimate FDR using PIPs for FABIO and FOCUS. Specifically for the local FDR approach, we first calculated the local FDR for all the genes, which is effectively 1-PIP. We then sorted genes based on these local FDRs from small to large. For each gene in turn, we estimated FDR for that gene by aggregating the local FDRs from the first gene up to that gene in the sorted list. Afterwards, we identified the cutoff value of PIP/local FDR that corresponds to a targeted FDR threshold of 0.05. This is the significant threshold we use that corresponds to an estimated FDR of 0.05.

Hopefully the above explanations have addressed your comment, “the threshold should be chosen using the same method (e.g., q-value < 0.05),”. Indeed, we did not "use this very high type-I error as the threshold for determining power,", but rather, we used the p-value threshold corresponding to the same false discovery rate (FDR) for determining power, ensuring a fair comparison across methods. By using the same FDR threshold of 0.05, we ensured a fair power comparison across methods, particularly since frequentist and Bayesian approaches output different statistics (p-values vs. PIPs). We suspect that our initial description of the FDR procedure may have been insufficient, potentially leading to the misunderstandings mentioned above. To clarify and address this, we have substantially enhanced the Materials and Methods section to provide additional details (see **lines 691-714 on pages 34-35**).

Finally, regarding your comments on FOGS, it was indeed designed to have more power when a larger number of SNPs exhibit non-zero effects on gene expression. To show this, we have included additional simulations to explore a non-sparse setting where many causal genes were present (one in each of the 50 LD blocks) and a substantial number of SNPs for the causal genes had non-zero effects on gene expression (100% of cis-SNPs). In this non-sparse scenario, consistent with the FOGS manuscript, the method produces well-calibrated type I error control (**Figure S2c, “50 genes; 100% SNPs”**). In this case, the power and AUC for FOGS also becomes comparable to FABIO and FOCUS (**Figure S6b and S6c**). However, when the number of causal genes decreases (e.g., one causal gene in 10 of the 50 LD blocks, with no causal genes in the remaining 40 blocks), even with 100% of cis-SNPs showing non-zero effects, type I error from FOGS is not well controlled (**Figure S2c, “10 genes; 100% SNPs”**). Furthermore, when only a small number of SNPs have non-zero effects (1% of cis-SNPs), even if there are many causal genes (one per LD block), FOGS fails to provide calibrated type I error control (**Figure S2c, “50 genes; 1% SNPs”**). Compared to all the sparse scenarios covered in our previous submission, the deficiency in power between FOGS and FOCUS or FOGS and FABIO determined by a true FDR threshold of 0.05 was smaller in these two less-sparse scenarios (**Figure S6b “10 genes; 100% SNPs” and “50 genes; 1% SNPs”**, respectively). In general, FOGS tends to have better performance in type-I error control and power in non-sparse scenarios of causal genes and causal SNPs, due to its model specification. These new results are shown in Supplementary Figures S2c and Figure S6, with details provided in the updated Results section (**lines 208-210 on page 10** and **lines 268-273 on pages 13-14**).

Sufficiency of simulation replications

As other reviewers may also point out, the number of simulation replications is only 20, which may not be sufficient for evaluating these three methods. I believe 100 replications should be performed for the main cases (e.g., those shown in Figures 2 and 3).

Thank you for your comment. We previously used 20 replications following earlier TWAS method papers (e.g., Li et al., 2021, which used 20 replications). Following your suggestion, we have increased the number of simulation replications from 20 to 100 for all alternative simulation settings in the revised manuscript. This makes the replications comparable to those used in the original FOCUS (50 replications) and FOGS (120 replications) papers. The results remain consistent with the increased number of replications, showing minimal changes in power at a true FDR threshold of 0.05: the average of absolute change rates was 2.12% for FABIO, 2.43% for FOCUS, and 2.35% for FOGS. Similarly, we observed minor changes in AUC: the average of absolute change rates was 3.05% for FABIO, 2.92% for FOCUS, and 2.67% for FOGS. Overall, the changes in both power and AUC were negligible (<5%), and the ranking of the three methods remained the same with the increased number of replications. The updated results for power and AUC are reflected in the revised **Figure 3**, and **Figures** **S4-S6**.

Addressing GReX correlation across the chromosome?

FABIO utilizes traditional GReX modeling, and the process of predicting SNP weights only uses LD information from the cis-region. In other words, although gene expression may have many variants with strong trans-regulatory effects (trans-eQTL), this trans-eQTL information is not utilized in predicting gene expression, resulting in LD-wise uncorrelated predicted gene expression. Therefore, my initial view is that although FABIO processes all genes on a chromosome simultaneously, it does not leverage GReX correlations among genes between LD blocks. Although these predicted genes are independent, analyzing them in the same model can utilize prior distribution information to control FDR, which I believe is an advantage of FABIO.

Thank you for your comment. Yes, similar to existing TWAS fine-mapping methods such as FOCUS and FOGS, FABIO utilizes the traditional GReX modeling framework, building gene expression prediction models using only cis-SNPs. These cis-SNPs are correlated due to linkage disequilibrium (LD), with correlation decaying as the distance between them increases. Consequently, the constructed GReX displays correlation not only within LD blocks but also between neighboring LD blocks, though the correlations decrease with increasing distance. To further illustrate this, we have performed two new lines of analysis on 1,568 genes from 133 distinct LD blocks on chromosome 1.

First, we calculated the pairwise correlation of GReX for four distinct groups of gene pairs: those within each LD block, between two adjacent LD blocks, between two distant LD blocks, and between an LD block on chromosome 1 and one on chromosome 2, with the latter serving as a baseline. As expected, we observed a clear decreasing trend in GReX correlation across these groups. Notably, the correlations between two adjacent LD blocks and between two distant LD blocks remained substantial—3.88 and 1.47 times higher, respectively, than the baseline correlation between LD blocks on different chromosomes (**Figure S13**). This suggests that GReX correlation extends beyond individual LD blocks, although with diminishing magnitude over increasing distance.

Second, for all six binary traits examined in our real data analysis, we computed the proportion of phenotype variance (R^2^) explained by GReX using linear regression in two ways. First, we included the GReX for all genes across 133 LD blocks on chromosome 1 in the model and computed the R^2^. Second, we included the GReX for all genes within each LD block, computed the R^2^ for each block, and then summed the R^2^ values across blocks. Intuitively, if GReX correlation between LD blocks is negligible, the R^2^ values obtained from these two approaches should be identical. However, we found that the average R^2^ computed in the first way is 1.38% (±0.49%) while that the average R^2^ computed in the second way is 1.74% (±0.52%), with a 26.1% increase due to GReX correlation between LD blocks. This highlights the importance of modeling GReX correlation between LD blocks, as is done in FABIO.

We have included the above two lines of evidence in the updated Discussion section (**lines 504-524 on pages 25-26**), with results displayed in the updated **Figure S13**.

Optimal tuning parameter selection

FABIO is a variable selection method, which belongs to the same category as lasso and SuSiE. For lasso and SuSiE, the penalty parameter lambda and the number of single effects L are the main tuning parameters that control their performance, respectively. In FABIO, the proportion of non-zero effects pi is a crucial tuning parameter. In the supplementary materials, the authors claim that they assume log pi follows a uniform distribution on U(log(1/p),0), while traditional methods tend to assume pi follows a beta distribution, adjusting a and b (the two parameters of the beta distribution) to control the mean and variance of pi. Can the performance of FABIO be improved by modifying the prior distribution of pi if the users have prior knowledge about the approximate proportion of important genes on a chromosome?

Thank you for your comment. Following your comment, we have incorporated the beta distribution as an additional prior choice for pi in the FABIO software, allowing users to adjust the parameters a and b by themselves. Details are provided in the updated Methods section (**lines 581-582 on pages 29**) and the updated Supplementary Text (**lines 13-15 on page 1**)

We applied FABIO using either the uniform prior on log pi or this new beta prior on pi in simulations. For the beta prior, we tested different combinations of a and b to exam how the expectation and variance of pi influence model performance. In the baseline simulation settings, when we set the prior expectation of pi to the truth, we did not observe apparent difference between our previous prior and the new beta prior of pi regardless of the prior variance of pi (i.e. a=1.1 and b=99; a=0.11 and b=9.9; a=11 and b=990). However, as expected, we did find that changing the prior expectation of pi (a=1.1 and b=99; a=1.1 and b=9.9; a=1.1 and b=999) did affect performance, with the prior expectation closer to truth yielding better performance. This suggests that prior knowledge can improve model performance but may not outperform our default prior on pi. These new simulation results are shown in **Figure S9** and summarized in detail in the updated Results section (**lines 307-309 on page 16**).

We further applied FABIO with the beta prior to the six disease traits in our real data application. Based on our observation in the simulations that the prior expectation, but not the prior variance, of pi influences performance, we fixed the parameter ***a*** at 1.1 and estimated the parameter ***b*** for each disease trait by assuming one causal gene per risk region (defined in Table 1). We found that FABIO with new beta prior yielded fewer signals inside the risk regions compared to the previous default prior on pi, as our input parameters of the beta prior may not reflect the unknown truth of the causal gene density. These results are summarized in **Table S1** as well as in the updated Results (**lines 352-353 on page 18**).

Software availability and documentation

I noticed that the software for FABIO has been made publicly available with a relatively detailed tutorial. I suggest that the authors prominently highlight this point in the manuscript (<https://superggbond.github.io/FABIO/>). I also suggest adding a short paragraph somewhere to briefly introduce how to use it, what inputs are, and which (important) tuning parameters are recommended to specify manually.

Thank you for your comment. Following your comment, we have added a sub-section in the updated Materials and Methods (**lines 816-835 on pages 39-40**) to briefly introduce how to use FABIO, what inputs are, and the recommended setting for the tuning parameters. This is listed below for your convenience.

**Software overview**

FABIO is implemented in an R package and is freely available at <https://github.com/superggbond/FABIO/>. FABIO requires an individual-level predicted GReX file of the TWAS cohort of study (generated using standard methods like PrediXcan and BSLMM) in either plain text format or gzipped text format, which has *m* (number of genes) rows and *n* (sample size) + 1 columns. The gene names serve as the first column, following *n* columns where each column represents GReX of genes for an individual. If the users only have genotypes from an individual-level GWAS in PLINK format, FABIO also provides a function to create the predicted GReX file using our prebuilt BSLMM gene expression prediction models derived from the GEUVADIS eQTL panel, the same eQTL panel used in our real data applications. FABIO also requires the binary phenotypes for the same cohort of *n* individuals in a plain text format, using 1 to indicate the case and 0 to indicate the control. The order of the binary phenotypes should be consistent with the column order of the predicted GReX file. A key parameter in the model is $\pi$, which represents the proportion of genes with non-zero effects. We present two prior options for $\pi$: a uniform prior on log π, which requires no input of $\pi$ from the users, and the initial $\pi$ will be the proportion of genes passing the single-gene tests based on the input predicted GReX file; or a beta prior $\pi\sim Beta(a,b)$, where users can choose the values of a and b if they have prior knowledge to determine the prior expectation and the variance of $\pi$. At the end, FABIO outputs the TWAS fine-mapping results as a table with three columns: the names of the genes, the PIPs of the genes, and the FDR estimate for each gene corresponding to the PIP value.

**Comments From Reviewer #2:**

In the manuscript under consideration, the authors presented a novel method, FABIO, using a Bayesian additive model to fine map genes in a TWAS framework. The mathematical formulation of the method is clearly presented. Simulations and real data analyses are professionally done. Although I think it is a great piece of work, there are some aspects may need to be clarified and explained more intuitively. Here are my comments for the authors’ considerations.

Thank you for your positive and constructive comments. Our detailed responses to your major and minor comments are listed below.

First, I feel that the idea of using a linear combination of GReX to predict phenotype for fine mapping a little bit counter intuitive. The default reason why fine mapping is needed is that the genes are correlated to each other, therefore better modeling of such correlations are required. However, the model just adds all genes (GReX) up, despite the potential problem of collinearity. Intuitively, the models that explicitly taking correlation structure (e.g., the pair-wise correlation matrix, analogue to the LD matrix of all variants in GWAS fine-mapping) into account may be the right solution. I didn’t mean the presenting method is total wrong. At least some intuitive interpretation is needed here.

Thank you for your comments. We apologize for not being clear on our model in the previous submission. Our model does not simply add all genes (GReX) up. Instead, we explicitly model their correlation structure using a probit modeling framework where all GReX are included as covariates. That is, the weights in the linear combination of GreX are automatically inferred from the model to account for their correlations. As a result, the pair-wise GReX correlation matrix is used in the fitting algorithm. Following your comment, we have included additional information to the updated Methods section to highlight this important feature of FABIO in modeling GReX correlations (**lines 570-572 on page 28**).

Second, there is a lack of details on how the cutoff of the methods are set to ensure a fair comparison between the three methods. It has been repetitively stated that FDR = 0.05 is used without specifics on how this FDR is calculated. However, I can’t figure out a default way to calculate FDR in the present framework with PIP. This is an important procedure that has not been presented.

Thank you for your comment. We apologize for not being clear on the cutoffs we applied. We calculated FDR in two different ways, corresponding to the terminologies “true FDR”, which was used in simulations, and “estimated FDR”, which was used in both simulations and real data applications. To compute the true FDR, in the simulations, we first divided 100 simulation replicates into five groups, with 20 replicates per group. In each group, we obtained the test statistics from each method for all genes across the 20 replicates, either in the form of p-values or PIPs. For each method in turn, we sorted the test statistics from the most significant (i.e. the smallest p-value or the largest PIP) to the least significant (i.e. the largest p-value or the smallest PIP). For each ordered test statistic in the sorted list, we counted the number of false positives detected above the threshold defined by this specific test statistic. The number of false positives, divided by the total number of genes called significant at that threshold, is FDR. We then determined threshold that corresponds to an FDR of 0.05, based on which we further calculated power. Power is calculated as the number of true positives above the threshold in the sorted list divided by the total number of causal genes. Afterwards, we obtained the average power across five groups to minimize stochasticity.

Because we do not have the ground truth, we cannot compute “true FDR” in the real data. Instead, we estimated FDR using different approaches: the Benjamini-Hochberg approach to estimate FDR using p-values from FOGS, and the local FDR approach (Efron 2007) to estimate FDR using PIPs for FABIO and FOCUS. Specifically for the local FDR approach, we first calculated the local FDR for all the genes, which is effectively 1-PIP. We then sorted genes based on these local FDRs from small to large. For each gene in turn, we estimated FDR for that gene by aggregating the local FDRs from the first gene up to that gene in the sorted list. Afterwards, we identified the cutoff value of PIP/local FDR that corresponds to a targeted FDR threshold of 0.05. This is the significant threshold we use that corresponds to an estimated FDR of 0.05.

To avoid confusion, we now clearly state that we use the true FDR threshold of 0.05 in the simulations since we know the ground truth, and rely on the estimated FDR threshold of 0.05 in the real data applications. The details of how we calculate the true FDR or estimate FDR have been included in the updated Methods section (**lines 691-714 on pages 34-35**).

**minor comments:**

The explanation of Figure 1 and the first few paragraphs in Results are redundant to existing texts in Methods. It appears to me that the paper was originally prepared in a format that Results show up earlier leaving Methods to the end. Better flow aligning to PLoS Genetics may be required.

Thank you. Following your comment, we have moved the Materials and Methods section to the end after the Discussion section in our updated manuscript. We have also streamlined the first few paragraphs in the Results section to avoid redundancy with Figure 1 (**lines 151-157 on page 8**).

The comparison to other methods could be more thorough. For instance, there are two newer papers published in Nat Genet on this topic (including the one published from the same group of this paper). It may be nice to carry out some comparisons.

Thank you. Following your comment, we have included GIFT and cTWAS into comparisons in both simulation and real data application. Both methods also use a multiple linear regression framework and do not directly model the binary nature of disease traits. In addition, both GIFT and cTWAS are computationally demanding: it takes about three days for either method to analyze on a relatively small LD block with <10 genes and over a week on a moderate-sized LD block with >20 genes. This is in direct contrast to the methods we previously compared: for example, it took FABIO, FOCUS and FOGS an average of 3.1, 1.5 and 9.2 hours to analyze all 50 LD blocks, respectively.

Due to the heavy computational demand of these two methods, we limited comparisons to the baseline simulation setting. In the simulations, as expected, FABIO achieved the best performance among the five methods, regardless whether using the true FDR of 0.05 (**Figure S10a**), or AUC based on ROC curves (**Figure S10b**). In the real data, due to the heavy computational demand of GIFT and cTWAS, we again restricted the comparison to a subset of LD blocks that contain no more than 20 genes. Across traits, we analyzed 8,411 genes from 1,259 unique LD blocks. We observed that FABIO identified the largest number of gene candidates lied in the GWAS or TWAS risk regions compared to the other methods including GIFT and cTWAS (**Table S2**).

These results are shown in Figure S10 and Table S2 and summarized in the updated Results (**lines 309-313 on page 16** and **lines 360-362 on page 18**).

**Comments From Reviewer #3:**

This study presents a novel method to prioritize causal genes by integrating GWAS and eQTL studies. It is novel in two aspects. First, it is designed for binary traits with a probit model. Second, it accounts for all genes on one chromosome. The authors evaluated the performance of the new method (FABIO) with simulated date and compared its performance to two existing methods (FUSION and FOGS). The performances of the three methods were also compared in the applications to six binary traits in the UK Biobank dataset. This manuscript is very well-written. It is very easy to follow, including the methodological details. With various simulation scenarios (e.g., case/control ratio, phenotypic variance explained, number of causal SNPs, and number of causal genes), the authors presented convincing evidence that FABIO has well-controlled false positive rates and much better power than the other two methods. In the application to real data, FABIO narrowed down to the smallest number of genes while identifying genes in the likely risk regions, defined based on GWAS or TWAS significant signals. The findings from FABIO are more robust to down-sampling. Gene set and pathway enrichment analysis further supported the biological relevance of the candidate genes fine-mapped by FABIO. Lastly, FABIO has acceptable computational efficiency for biobank-scale dataset. Overall, this study developed an outstanding method and package for the field of GWAS. It is likely to be widely used in GWAS of binary traits. It is quite a pleasure to read this manuscript, which is informative and easy to follow. I support the publication of the manuscript in its current form. I do have one suggestion for the author’s consideration.

Thank you for your positive and constructive comments as well as the compliments. Our detailed responses to your major comments are listed below.

**Major comment:**

The very poor performance of one of the comparison method, FOGS, in both simulation and application is kind of surprising. It will be useful to readers if the authors could discuss or propose some explanations. Does it have to do with the specific simulation approaches? Or with the binary traits, instead of quantitative traits?

Thank you for your comment. Following your comment, we have carefully examined FOGS and found that FOGS was designed to have more power when a larger number of SNPs exhibit non-zero effects on gene expression. To show this, we have included additional simulations to explore a non-sparse setting where many causal genes were present (one in each of the 50 LD blocks) and a substantial number of SNPs for the causal genes had non-zero effects on gene expression (100% of cis-SNPs). In this non-sparse scenario, consistent with the FOGS manuscript, the method produces well-calibrated type I error control (**Figure S2c, “50 genes; 100% SNPs”**). In this case, the power and AUC for FOGS also becomes comparable to FABIO and FOCUS (**Figure S6b and S6c**). However, when the number of causal genes decreases (e.g., one causal gene in 10 of the 50 LD blocks, with no causal genes in the remaining 40 blocks), even with 100% of cis-SNPs showing non-zero effects, type I error from FOGS is not well controlled (**Figure S2c, “10 genes; 100% SNPs”**). Furthermore, when only a small number of SNPs have non-zero effects (1% of cis-SNPs), even if there are many causal genes (one per LD block), FOGS fails to provide calibrated type I error control (**Figure S2c, “50 genes; 1% SNPs”**). Compared to all the sparse scenarios covered in our previous submission, the deficiency in power between FOGS and FOCUS or FOGS and FABIO determined by a true FDR threshold of 0.05 was smaller in these two less-sparse scenarios (**Figure S6b “10 genes; 100% SNPs” and “50 genes; 1% SNPs”**, respectively). In general, FOGS tends to have better performance in type-I error control and power in non-sparse scenarios of causal genes and causal SNPs, due to its model specification. These new results are shown in Supplementary Figures S2c and Figure S6, with details provided in the updated Results section (**lines 208-210 on page 10** and **lines 268-273 on pages 13-14**).

**References**

Weissbrod, Omer, et al. "Accurate liability estimation improves power in ascertained case-control studies." *Nature methods* 12.4 (2015): 332-334.

Hujoel, Margaux LA, et al. "Liability threshold modeling of case–control status and family history of disease increases association power." *Nature genetics* 52.5 (2020): 541-547.

Zhou, Xiang, Peter Carbonetto, and Matthew Stephens. "Polygenic modeling with Bayesian sparse linear mixed models." *PLoS genetics* 9.2 (2013): e1003264.

Efron, Bradley. "Size, power and false discovery rates." (2007): 1351-1377.

Li, Binglan, et al. "Tissue specificity-aware TWAS (TSA-TWAS) framework identifies novel associations with metabolic, immunologic, and virologic traits in HIV-positive adults." *PLoS genetics* 17.4 (2021): e1009464.
