# Supplementary material for: FABIO: TWAS fine-mapping to prioritize causal genes for binary traits
Source: PLoS Genet. 2024 Dec 2;20(12):e1011503. doi: 10.1371/journal.pgen.1011503 (PMC11649093; doi:10.1371/journal.pgen.1011503)
Supplement: Attachment — Submitted filename: response_letter_2nd_revision.docx [file pgen.1011503.s019.docx]

**Comments From Reviewer #1:**

I am pleased and grateful that you have provided a comprehensive response, and have modified your method using one of my suggestions. I believe you have appropriately incorporated the changes.

Thank you.

However, I still have a minor question regarding the calculation of the true FDR and local FDR. I am grateful that you can address, as they may be due to my limited knowledge in this area.

1. When calculating the true FDR in the simulations, why did you choose to divide the simulations into 5 groups?

Thank you. We used this procedure to minimize stochasticity and improve the stability of the results, following previous work (e.g. Yuan et al., 2020; Song et al., 2020; Song et al., 2021; and Liu et al., 2024). Following your comment, we have included these additional citations in the updated Methods section (**line 703 on page 34**).

2. I am very interested in your approach to adjusting the PIP based on Efron's method. This could potentially benefit all current work based on SuSiE. After reading Efron's paper, I understand that this method requires estimating the density function of a mixture model and pre-specifying some tuning parameters like p0 (equation 2.13). While Efron provided some guidance on how to estimate these, I was hoping you could share a more detailed pipeline on how you applied this method in your work (e.g., whether it is able to leverage any existing R packages to facilitate the estimation process).

Thank you. The procedure outlined in Efron's paper, such as estimating the density function of a mixture model and pre-specifying tuning parameters like p0 (see Equation 2.13), is designed for converting z-scores or p-values into local FDR values. In contrast, we directly convert PIP to “local FDR” based on its definition: local FDR for a gene is defined as the probability that the null hypothesis is true given the observed data for this gene, while PIP represents the posterior probability that the gene is truly causal. Therefore, we can convert PIP to local FDR as 1-PIP. This was previously explained in the Methods section: “Specifically, for the local FDR approach, we first calculated the local FDR for all genes, which is effectively 1 - PIP.” (**lines 709-710 on pages 34-35**) Due to the simplicity of the procedure, no additional software package is needed. Following your comment, we have included additional citations (e.g. Liu et al., 2024 and Gao & Zhou, 2024) to refer readers to this established procedure.

3. I would like you to help me understand that the term "local" in the local FDR context refers to a statistical concept, and not a genetic locus or a block in the block-wise GReX estimation.

Thank you. In the context of local FDR, the term “local” refers to a statistical concept and is not related to a genetic locus or a block in GReX estimation. Specifically, “local FDR” represents the probability that a null hypothesis is true given the observed data at a given test statistic (hence it’s “local”).

**References**

Yuan, Zhongshang, et al. "Testing and controlling for horizontal pleiotropy with probabilistic Mendelian randomization in transcriptome-wide association studies." *Nature communications* 11.1 (2020): 3861.

Song, Yanyi, et al. "Bayesian shrinkage estimation of high dimensional causal mediation effects in omics studies." *Biometrics* 76.3 (2020): 700-710.

Song, Yanyi, et al. "Bayesian hierarchical models for high‐dimensional mediation analysis with coordinated selection of correlated mediators." *Statistics in medicine* 40.27 (2021): 6038-6056.

Liu, Lu, et al. "Conditional transcriptome-wide association study for fine-mapping candidate causal genes." *Nature Genetics* 56.2 (2024): 348-356.

Gao, Boran, and Xiang Zhou. "MESuSiE enables scalable and powerful multi-ancestry fine-mapping of causal variants in genome-wide association studies." *Nature Genetics* 56.1 (2024): 170-179.
